# Multifaceted design optimization for superomniphobic surfaces

**DOI:** 10.1126/sciadv.aav7328

**Published:** 2019-06-21

**Authors:** J. R. Panter, Y. Gizaw, H. Kusumaatmaja

**Affiliations:** 1 Department of Physics, Durham University, South Road, Durham DH1 3LE, UK.; 2 Procter and Gamble Co., Winton Hill Business Center, 6210 Center Hill Avenue, Cincinnati, OH, USA.

## Abstract

Superomniphobic textures are at the frontier of surface design for vast arrays of applications. Despite recent substantial advances in fabrication methods for reentrant and doubly reentrant microstructures, design optimization remains a major challenge. We overcome this in two stages. First, we develop readily generalizable computational methods to systematically survey three key wetting properties: contact angle hysteresis, critical pressure, and minimum energy wetting barrier. For each, we uncover multiple competing mechanisms, leading to the development of quantitative models and correction of inaccurate assumptions in prevailing models. Second, we combine these analyses simultaneously, demonstrating the power of this strategy by optimizing structures that are designed to overcome challenges in two emerging applications: membrane distillation and digital microfluidics. As the wetting properties are antagonistically coupled, this multifaceted approach is essential for optimal design. When large surveys are impractical, we show that genetic algorithms enable efficient optimization, offering speedups of up to 10,000 times.

## Teaser

Versatile computational tools are developed to solve the design optimization challenge for structured nonwetting surfaces.

## INTRODUCTION

Superomniphobic surfaces show physical micro- or nanotexturing that enable even low–surface tension liquids to remain suspended atop a vapor-filled surface structure. This vapor-suspended state is prized for its liquid-shedding abilities, enabling high droplet mobility and low viscous drag (*1*). These surfaces have considerable potential to be transformative across a broad array of applications. These range from tackling current global-scale crises, via sustainable technologies for water purification (*2*, *3*) and antimicrobial surfaces in biomedicine (*4*, *5*), through everyday applications such as antifingerprint coatings (*6*) and packaging designed to reduce food waste (*7*), to digital microfluidics as a versatile biological and chemical technology (*8*).

Two promising textures aimed to enable these technologies are the reentrant and doubly reentrant geometries. Naturally occurring examples of these structures have been shown to imbue the cuticle of the springtail arthropod (Collembola) with superolephobic properties even for highly wetting, pressurized liquids, while further exhibiting abrasion-resistance and antimicrobial abilities (*9*–*12*). Recent breakthroughs in microfabrication techniques have also allowed these reentrant and doubly reentrant structures, as well as more complex textures, to be produced with micrometer-scale resolution, including the use of three-dimensional (3D) printing technology, fluidization of polymer micropillars, and lithographic methods (*13*–*16*).

Despite these highly versatile techniques, a large obstacle still exists to widespread development: It is not known how to design the surface structures to enable optimal performance in real-world applications. Successful superomniphobic designs must exhibit three key wetting properties: (i) a low contact angle hysteresis (CAH) to maximize liquid mobility (*17*), (ii) a high critical pressure, the maximum sustainable pressure at which the superoloephobic state is stable (*18*), and (iii) a high energetic barrier to failure, in which liquid infiltrates the surface texture and the high liquid mobility is lost (*19*).

The complex surface designs mean that both computational and experimental studies are expensive and time consuming to perform, and so are largely restricted in scope to considering only single wetting properties—never all three. This is highly problematic as the structural parameters of the design couple each wetting property, often antagonistically. For example, two effective ways to increase the critical pressure are to decrease the pillar-pillar separation and decrease the system scale (*17*). However, decreasing the separation results in an increase in the CAH (*20*), whereas decreasing the system scale decreases the energetic barrier to the wetting transition due to the liquid-vapor interfacial area decreasing in squared proportion. Although this threefold perspective has been introduced and advocated before [see, for example, (*17*)], the true lowest-energy failure mechanisms have never been incorporated.

This work overcomes the optimization challenges for superomniphobic wetting property design. We begin by developing computational strategies to systematically survey the effect of the structural parameters on the CAH, critical pressure, and minimum energy barrier individually. These methods are highly general and can be applied to any conceivable surface design. This leads to the discovery of previously unidentified mechanisms for the receding contact line and failure of the suspended state, as well as the development and validation of quantitative analytical models. We correct a number of inaccurate assumptions in prevailing models. In particular, we highlight the development of a capillary bridge model to replace the grossly inaccurate prediction for the optimal texture height in the critical pressure study.

To illustrate the importance of multifaceted optimization, we then consider two relevant example applications: water purification via membrane distillation and droplet-based digital microfluidics (Results and Discussions). Membrane distillation shows considerable potential as a sustainable, low-energy water purification technology, capable of extracting potable drinking water from highly contaminated water sources [see (*2*, *3*) for recent reviews]. A major challenge, however, is that oils readily foul the membranes, leading to a breakdown in device performance. Meanwhile, digital microfluidics is anticipated to enable reusable, reconfigurable, and material-efficient lab-on-chip devices (*8*). In this technology, the major challenge is that commonly used but low–surface tension solvents pin strongly to the surface, leading to drop immobilization and device failure (*21*). We find here that the doubly reentrant surface geometry is ideally situated to meet these challenges, as it is robust to pressure even for highly wetting or surfactant-contaminated liquids.

In such complex surface design featuring many antagonistically coupled wetting properties, we recognize that it is not always desirable to perform large-scale wetting property surveys. Thus, we develop a genetic algorithm to perform the simultaneous optimization with high efficiency, offering a speedup of up to 10,000 times. This versatile approach is highly complementary to recent innovations in complex surface microfabrication techniques (*13*–*16*), such that, together, we anticipate a transformative approach to surface design.

## RESULTS AND DISCUSSIONS

### Contact angle hysteresis

**Fig. 1:**
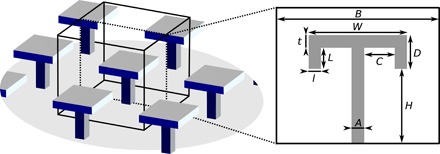
Simulation surface configuration. Illustration of the 3D simulation repeat unit (left), with 2D cross section showing labeled structural parameters (right).

We begin by simulating the liquid-vapor interface advancing and receding along a single row of surface structures (setup detailed in the Supplementary Materials), to obtain the macroscopic advancing and receding contact angles θ_a_ and θ_r_, respectively, and the CAH (CAH = θ_a_ − θ_r_). These simulated structures are shown in Fig. 1, in which structures of variable dimensions are arranged in a square array. Throughout, all dimensions shown in Fig. 1 are reported relative to the system size *B* and indicated with a subscript “r”. For example, the reduced cap width is *W*_r_ = *W*/*B*. Unless otherwise stated, *B* = 60 lattice spacings, with the cap thickness *t*_r_ and lip width *l*_r_ remaining fixed at 0.05. For the reentrant geometries, the lip depth is *L*_r_ = 0. Throughout, the microscopic contact angle θ_°_ = 60° is used as a representative contact angle for organic solvents wetting fluorinated surfaces [see, for example, (*22*)]. We also investigate the nonwetting case presented in the Supplementary Materials.

**Fig. 2:**
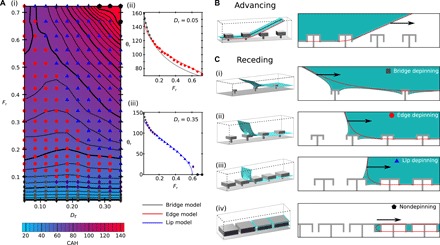
Quantification and mechanisms leading to the CAH for reentrant and doubly reentrant geometries at zero applied pressure. (**A**) (i) CAH dependence on both the area fraction *F*_r_ and total cap height *D*_r_. Symbols indicate the depinning mechanism upon receding, with purple diamonds indicating a hybrid mechanism. (ii and iii) Comparison of the bridge-, edge-, and lip-depinning receding models (solid lines, color-coded) against the simulated θ_r_ (data points); examples shown with varying *F*_r_ at fixed *D*_r_ = 0.05 and 0.35. The ±1° error bars in the simulation data are too small to be seen. (**B**) 3D visualization of the advancing liquid-vapor interface (shown in blue); the advancing direction is indicated by a black arrow. Black and red lines indicate the center and edge 2D cross sections that are also presented (right). (**C**) (i to iv) Visualizations of the major four receding mechanisms. The receding direction is indicated by black arrows.

We find that the CAH depends only on the area fraction *F*_r_ of the cap [*F*_r_ = (*W*/*B*)^2^], and the total cap height *D*_r_, as shown in Fig. 2A (i). Separate advancing and receding plots are shown in the Supplementary Materials, alongside available comparison with previous experimental measurements. As the liquid-vapor interface never impinges under the cap, the hysteresis is identical for both reentrant and doubly reentrant geometries.

Across the simulated parameter range, the same advancing mechanism is observed, as illustrated in Fig. 2B. In this, the advancing occurs when the angle of the approximatively planar liquid-vapor interface results in the liquid contacting the cap of the neighboring structure.

In contrast, the receding mechanism exhibits considerable variation across the parameter range and is therefore primarily responsible for the large variation in CAH observed in Fig. 2A (i). Overall, four dominant receding mechanisms are observed: bridge, edge, lip depinning, and a fourth nondepinning mechanism. Characteristic examples of these are shown in Fig. 2C (i to iv), respectively. The operative regions of each mechanism are labeled in Fig. 2A (i), indicated by gray squares, red circles, blue triangles, and black pentagons, respectively. The hybrid depinning mechanisms (purple diamonds) indicate the regions in which the dominant mechanisms smoothly interpolate. We now describe and model each of these receding mechanisms in turn.

For the lowest area fractions, at the point of receding, the three-phase contact line is pinned to the outermost pillar around the top perimeter of the cap, as shown in Fig. 2C (i). A capillary bridge is strained between the cap and bulk liquid, such that receding occurs at the point of bridge depinning. This has been observed experimentally, (*20*, *23*, *24*), and we are now able to quantitatively test the receding model proposed by Butt *et al.* (*20*, *24*). In this model, the receding liquid-vapor interface strains the capillary bridge parallel to the receding direction, thus tilting the bridge from the normal (detailed in the Supplementary Materials). The two suggested consequences of this are that the direction of the pinning force is tilted from the normal by π/2 − θ_r_/2 and that this force depends on the average contact angle $\bar{\theta }=\theta_{{}^{\circ}}+\pi /2-\theta_{r}/2$. However, to yield an accurate model for use with wetting liquids as in this work, we stress that the appropriate average contact angle to use is $\bar{\theta }=\theta_{{}^{\circ}}^{\text{max}}+\pi /2-\theta_{r}/2$, where $\theta_{{}^{\circ}}^{\text{max}}=\text{max}(\theta_{{}^{\circ}},\pi /2)$. This is because for wetting liquids, the maximum pinning force is achieved when the contact angle reaches 90°. With this modification, at the point of receding this microscopic pinning force balances the macroscopic force required to move the contact line, yielding

$$\text{tan}(\frac{\theta_{r}}{2})=\frac{2}{4W_{r}\alpha }$$ (1)

In the simplest model, it is assumed that at the point of depinning, the three-phase contact line closely follows the square cap perimeter of length 4*W*_r_. However, to reflect the actual contact line morphology, the shape parameter α is introduced. α is equal to 1 if the contact line is perfectly square and π/4 for a circle. As the shape parameter cannot be predicted a priori, it is treated as a fitting parameter. An example of this is shown in Fig. 2A (ii, gray curve and square points) for *D*_r_ = 0.05. Here, α = 0.861, reflecting the contact line deviating from perfectly square by depinning at the cap corners. This yields an average agreement between the simulation and model of 0.4° (average agreement for all *D*_r_ tested is 0.6° and maximum is 2°).

At large *D*_r_ and *F*_r_, a previously unidentified capillary bridge depinning mechanism is observed in which the bridge is strained between the cap edge and bulk liquid phase. This lip-depinning receding mechanism, as shown in Fig. 2C (iii), results in a substantial decrease in 𝜃_r_, demonstrated in Fig. 2A (iii, blue curve and triangular points). The model that we introduce is to approximate the receding interface as a capillary bridge pinned to the side of the cap, stretched parallel to the receding direction (detailed in the Supplementary Materials). As with the bridge-depinning model, however, we must account for the average contact angle around the contact line, yielding

$$\text{cos}(\frac{\theta_{r}}{2})=\frac{2(W_{r}+D_{r})\alpha }{2}$$ (2)

The accuracy of this model is demonstrated in Fig. 2A (iii), at *D*_r_ = 0.35, for which α = 0.887. This yields an average deviation between the simulation and model of 1° (average deviation for all *D*_r_ tested is 2° and maximum is 6°).

This lip-depinning model also predicts the existence of systems in which a receding contact angle no longer exists. In these extreme cases of the lip-depinning mechanism, because depinning is not able to occur, a droplet caused to move across the surface would leave a trail of suspended liquid trapped between the caps. We observe this predicted nondepinning mechanism in simulations, indicated by black pentagons in Fig. 2A (i and iii). This nonreceding case is illustrated in Fig. 2C (iv).

At intermediate area fractions and lip depths, the depinning mechanism is no longer capillary bridge mediated. Instead, the edge-pinned receding mechanism is observed, as shown in Fig. 2C (ii). Here, the interface maintains approximately the same morphology as it depins laterally from the edge of the cap. Thus, we are able to analyze the energetic change of sliding the interface laterally by a small distance to obtain the angle at which receding becomes energetically favorable—the receding angle. This is derived in the Supplementary Materials. Taking into account the liquid receding from the cap top and edges and top surface of the microchannel

$$\text{cos}\theta_{r}=(W_{r}+2D_{r})\text{cos}\theta_{{}^{\circ}}+W_{r}-1$$ (3)

This represents a generalization of previous edge-depinning models (*25*, *26*), in which by taking account of the liquid receding from the cap sides, we are now able to describe the edge-depinning mechanism accurately for wetting liquids. This is demonstrated in Fig. 2A (ii, red curve and circular points). Without any fitting parameters, the average deviation between the model and simulation results is 2° (average deviation for all *D*_r_ tested is 3° and maximum is 7°).

### Critical pressure

**Fig. 3:**
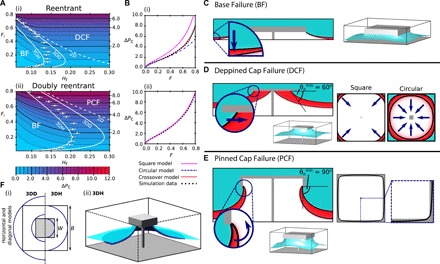
Critical pressure analysis for reentrant and doubly reentrant geometries. (**A**) Contour plots of Δ*P*_c_ variation with *F*_r_ and *H*_r_ for reentrant (i) and doubly reentrant (ii) geometries. Data points mark the critical height at which the failure mechanism switches from Base Failure (BF) to Depinned Cap Failure (DCF) or Pinned Cap Failure (PCF), and error bars indicate the uncertainty in this height due to the diffuse interface width. Solid and dashed white lines show the critical height based on the capillary model and 2D model, respectively. (**B**) Model fits to Δ*P*_c_ of the Cap Failure mechanisms at *H*_r_ = 0.25 for reentrant (i) and doubly reentrant (ii) geometries. (**C** to **E**) The three failure mechanisms shown in 3D, with associated diagonal cross sections. Critical pressure liquid morphologies are shown in blue, the vapor phase is shown in white, and the interface is indicated with a black solid line. Red regions show how the unstable meniscus evolves upon increasing Δ*P* above Δ*P*_c_. (**D** and **E**) Under-cap views, highlighting the shapes of the contact lines at the critical pressure. (**F**) Details of the 3D horizontal (3DD) and 3D diagonal (3DH) capillary bridge models used, showing the inner and outer circumferences (blue) against the system configuration. The 3D illustration compares the simulated liquid-vapor interface (light blue) to the horizontal capillary model (dark blue).

Unlike with the CAH, the critical pressure is sensitive to whether the surface geometry is reentrant or doubly reentrant. Throughout, the critical pressure shown Δ*P*_c_ is referenced with respect to the pressure γ_lv_/*B*. Here, we find that Δ*P*_c_ is only dependent on the area fraction *F*_r_ and the pillar height *H*_r_. Although Δ*P*_c_ is affected by the presence of a doubly reentrant lip, Δ*P*_c_ does not depend on the precise lip depth *L*_r_. The critical pressure dependencies on *F*_r_ and *H*_r_ are shown for the reentrant and doubly reentrant structures in Fig. 3A (i and ii), respectively. For both structural types, these dependencies change across the parameter space because of the presence of two different pressure-induced failure mechanisms: Base Failure and Cap Failure.

For a given area fraction *F*_r_, at low pillar heights, the Base Failure mechanism is operative, as illustrated in Fig. 3C. In this, the suspended state fails because the sagging liquid-vapor interface touches the base of the system while the three-phase contact line remains pinned to the bottom of the cap. By increasing the pillar height, Δ*P*_c_ is increased.

However, at *H*_r_ above the critical height *H*_c_, the Cap Failure mechanism becomes operative. Here, at the critical pressure, the system can no longer simultaneously support the uniform mean curvature of the liquid-vapor interface and the contact line morphology. For the reentrant geometry, this results in the contact line depinning and sliding inward, as shown in Fig. 3D. For the doubly reentrant geometry with a thin lip width *l*_r_, this results in the liquid-vapor interface ballooning outward while the contact line remains pinned, as shown in Fig. 3E. In both of these cases, increasing the pillar height further now results in no change to Δ*P*_c_.

Therefore, at fixed *F*_r_, the maximum Δ*P*_c_ occurs for *H*_r_ ≥ *H*_c_, in the Cap Failure region. However, it is detrimental for design performance if the height is increased above the critical height, as this mechanically weakens the structure without increasing Δ*P*_c_ (*27*, *28*). The optimum pillar height is therefore *H*_r_ = *H*_c_, which defines the Base Failure–Cap Failure boundary. We therefore focus on discussing the critical pressure due to the Cap Failure mechanisms, before analyzing the critical height.

#### Depinned Cap Failure for reentrant geometries

To understand how Δ*P*_c_ is influenced by the area fraction (or alternatively the cap width *W*_r_) in the depinned Cap Failure mechanism, we begin by examining the rudimentary model proposed by Tuteja *et al.* (*17*, *29*). In this

$$\Delta P_{c}=\frac{4\alpha \text{sin}\theta_{{}^{\circ}}}{\frac{1}{W_{r}}-\alpha W_{r}}$$ (4)

For convenience, we incorporate the shape parameter α, the same parameter as defined previously in the CAH section, to unify the critical pressure models on circular (α = π/4) and square (α = 1) geometries. To rationalize this model, at Δ*P*_c_, the pinning force of the contact line balances the force due to the pressure over the area between the pillars (*17*, *29*). Two key assumptions are made. First, the contact line is supposed to follow the cap edge, while second, the contact angle around the contact line is presumed to be uniform and equal to θ_°_. We test this model in Fig. 3B (i). The square-cap model is observed to fit the simulation results very poorly, overestimating the critical pressure by between 26% and 95% in the tested range 0.016 ≤ *F*_r_ ≤ 0.8. If instead, a circular contact line model is used, we find that this agrees with the simulation data up to moderate area fractions (*F*_r_ < 0.6). Overall, by observing the contact line shape obtained through simulations, as shown in Fig. 3D, we find that the contact line varies in morphology, from circular at low *F*_r_ to approximately square at high *F*_r_.

We now develop a more sophisticated model, capable of accurately describing the critical pressure for reentrant and doubly reentrant geometries, at all contact angles. Three modifications are introduced to Eq. 4. First, it cannot be assumed that the contact line follows the cap edge, leading to the introduction of *W*_r_′, the corrected reduced width: *W*_r_′ = *W*_r_ − *a*, where *a* is a parameter that describes the difference between the actual width that the contact line assumes and the width of the cap. Since *a* cannot be predicted a priori, we treat *a* as a second fitting parameter. For reentrant geometries and doubly reentrant geometries with θ_°_ > 90°, we anticipate *a* ≈ 0, due to contact line pinning on the outer edge. For doubly reentrant geometries with θ_°_ < 90°, we anticipate *a* ≈ 2*L*_r_, due to contact line pinning on the inner edge. Second, we propose that the shape parameter α varies continuously as a function of *W*_r_′ between the circular and square limits, such that $\alpha =\frac{\pi }{4}+(1-\frac{\pi }{4}){\left(W_{r}\prime \right)}^{x}$. The exponent *x* describes the strength of this crossover and is a second fitting parameter. Third, if the contact angle on the hydrophilic reentrant geometry is increased, the pinning force of the contact line is maximized at θ_°_ = 90°. For θ_°_ > 90°, the pinning force remains at this maximal value (shown in the Supplementary Materials). Thus, to generally describe the critical pressure on all reentrant geometries, we replace θ_°_ in Eq. 4 with the corrected contact angle $\theta_{{}^{\circ}}^{\text{min}}=\text{min}[\theta_{{}^{\circ}},90{}^{\circ}]$.

This crossover model is shown in Fig. 3B (i) to be in excellent agreement with the simulation data, yielding *a* = 0.023 and *x* = 6.7. As anticipated, *a* is small relative to the cap width and is of the order of the diffuse interface width (ϵ/*B* = 0.01). The large exponent *x* reflects the simultaneous change of both the perimeter and area of the contact line as the system crosses from a circular to square configuration.

#### Pinned Cap Failure for doubly reentrant geometries

Next, we consider the critical pressures of the doubly reentrant structure, as shown in Fig. 3B (ii). In the pinned Cap Failure region, the liquid-vapor interface is pinned to the inner cap edge, as shown in Fig. 3E. The doubly reentrant lip enforces an approximately square contact line across the entire range of *F*_r_ tested, such that excellent agreement between the simulations and the critical pressure model in Eq. 4 is achieved at *x* = 0, α = 1, and *a* = 0.080. This model is also successfully used for θ_°_ = 110° in the Supplementary Materials. As anticipated, within the uncertainty introduced by the diffuse interface, *a* ≈ 2*L*_r_ (where 2*L*_r_ = 0.1). However, the data in Fig. 3B (ii) are best described by replacing θ_°_ in Eq. 4 with 90°. In 2D, and for axisymmetric doubly reentrant wells, it is facile to show that the critical pressure occurs when the contact angle reaches 90° for thin lip widths *l*_r_ (*10*). We conclude here that this remains true, even for 3D, nonaxisymmetric caps. This therefore verifies the proposition that the doubly reentrant lip maximizes the critical pressure for any surface wettability (*16*).

#### Critical heights

At a given *F*_r_, the critical heights are obtained both analytically and through simulations by observing the maximum depth that the liquid-vapor interface sags under the pillar in the Cap Failure regions. If the pillar height *H*_r_ is equal to this sagging depth, the failure mechanism is simultaneously Base Failure and Cap Failure, thus defining the failure mode boundary and the critical height *H*_c_. The salient observation based on the simulated critical heights (white data points in Fig. 3A, i and ii) is that regardless of cap area fraction, the optimal pillar height is unexpectedly short and should never exceed ≈ 0.2*B*. We now rationalize this observation.

In many critical pressure models, a 2D circular-arc model is used to estimate the sagging height of the liquid-vapor interface

$$H_{c}=\frac{S_{r}}{2}\frac{1-\text{cos}\theta_{{}^{\circ}}^{\text{min}}}{\text{sin}\theta_{{}^{\circ}}^{\text{min}}}$$ (5)

where the separation *S*_r_ = (1 − *W*_r_) between horizontally adjacent pillars [see, for example, (*17*, *29*)], or $S_{r}=\sqrt{2}(1-W_{r})$ for diagonally separated pillars [see, for example, (*30*)]. The latter model is shown in Fig. 3A (i and ii, dashed white lines), and exemplifies the conclusion that, except at very high *F*_r_, a 2D estimation grossly overestimates the critical height. All currently manufactured, low-*F*_r_ structures relying on these 2D models are therefore substantially taller than necessary, which can be seen in (*18*) and (*31*), for example.

The actual, nonmonotonic critical height variation with *F*_r_, as shown in Fig. 3A (i and ii), can be rationalized by considering that in 3D, two principal radii of curvature characterize the liquid-vapor interface at each point. At low *F*_r_, the small contact line radius enforces a small, negative principal radius if curvature *R*_1_ on the liquid-vapor interface. Since the critical pressure is positive and proportional to 1/*R*_1_ + 1/*R*_2_, the second principal radius of curvature *R*_2_ must be smaller in magnitude than *R*_1_ and positive, resulting in a substantially reduced sag height compared to the 2D case. At large *F*_r_, however, observable for the reentrant geometry in Fig. 3A (i), the 2D model is recovered as the principal radius of curvature approximated by the circular arc (*R*_1_) and is considerably smaller than the second principal radius of curvature of the interface (*R*_2_). In this case, the interface shape becomes well approximated by the single radius of curvature *R*_1_.

We therefore recognize that the liquid-vapor interface is able to be modeled as a capillary bridge, for which we define the inner radius to contact the cap at an angle $\theta_{{}^{\circ}}^{\text{min}}$ and the outer radius to contact the simulation boundary with an angle equal to zero. Because the capillary bridge is axisymmetric, whereas the simulated system is square, there are two limiting cases of where the inner and outer radii contact the structure and simulation boundary, respectively, as shown in Fig. 3F (i). Either the inner and outer radii contact the central edges of the structure and simulation boundaries (the horizontal model, representing the minimum possible radii) or contact is made at the corners (the diagonal model, representing the maximum possible radii). Both potential capillary models are shown in Fig. 3A (i and ii, solid white lines). We reserve the detailed derivation to the Supplementary Materials.

In all cases, it is observed that the simulated critical heights are bounded by the horizontal and diagonal capillary bridge models. Furthermore, at low *F*_r_, the horizontal capillary model accurately predicts the critical height. For structures where the interface is pinned to the outer cap edge, namely, reentrant geometries and doubly reentrant geometries with θ_°_ > 90° (shown in the Supplementary Materials), the diagonal model is shown to closely predict the critical height at high *F*_r_. An illustrated comparison of the horizontal and diagonal models is shown in Fig. 3F (ii). Through this, we are able to successfully capture the critical height suppression at low *F*_r_ and maximum *H*_c_ at intermediate *F*_r_. We further validate the capillary bridge model in the Supplementary Materials by showing that this model is able to accurately reproduce an experimental interfacial profile from (*32*), whereas a circular arc model substantially overestimates the interfacial sagging height.

### Minimum energy transition mechanisms

#### Transition states and pathways

To design surfaces that maintain a suspended state in challenging environments, it is not sufficient to only understand how susceptible a surface design is to pressure. Failure can be initiated through a broad range of additional perturbations, such as flow (*33*), vibration (*34*), evaporation (*35*), condensation (*36*), droplet impact (*37*), changes to electric (*38*) or magnetic (*31*) fields, or thermal fluctuations at the nanoscale (*39*). In a real application, several perturbations will be present simultaneously, meaning that failure is unlikely to be initiated by only a single perturbation but instead via their combination. In fabricating a texture resistant to failure, it is therefore vital to understand this combined failure in the worst-case scenario, the minimum-energy pathway (MEP) by which the suspended state collapses. This is a steepest-descent pathway between two metastable states, in which the maximum energy along the path occurs at a saddle point (the transition state). The minimum energy barrier is the difference in energies between the suspended and transition states. This places a lower bound on the collapse energy barrier. If this barrier cannot be overcome by the perturbations applied to a candidate surface design, then the suspended state can be guaranteed to remain stable.

**Fig. 4:**
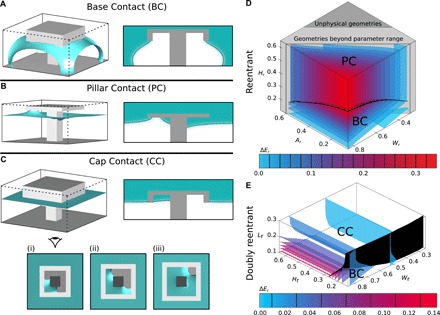
Reentrant and doubly reentrant transition state analysis. (**A** to **C**) 3D visualizations of the transition states of each transition pathway (liquid-vapor interfaces shown in blue) with associated diagonal cross sections (liquid-vapor interface outlined in black). (C) Under-cap views show the three CC transition state morphologies. (**D**) 3D contour plots showing the energy barrier Δ*E*_r_ of the lowest energy transition mechanism for the reentrant geometries. Each surface is a surface of constant Δ*E*_r_. The dividing surface between different transition mechanisms is shown in black. Impossible geometries with pillar widths *A*_r_ wider than the cap width *W*_r_ are shaded in dark gray. Geometries approaching this limit and requiring infeasibly large computational domains are shaded in light gray. (**E**) 3D contour plots showing the energy barrier Δ*E*_r_ of the lowest energy transition mechanism for the doubly reentrant geometries.

Through utilizing the Doubly Nudged Elastic Band algorithm (*40*), three transition pathways are found: Base Contact (BC), Pillar Contact (PC), and Cap Contact (CC), visualized in movies S1 to S3, respectively. From these, the transition state morphologies are surveyed using the gradient-squared method, each of which is shown in Fig. 4 (A to C). In the large-scale structural surveys, as we only wish to obtain the transition states and minimum energy barriers rather than the full paths, the gradient squared method is considerably more efficient that the full-pathway algorithm, as only a single minimization is required, as opposed to evolving a string of multiple systems across the landscape. These transition search algorithms are detailed further in the Supplementary Materials. We begin by discussing the qualitative characteristics of each before quantifying the suspended-to-collapsed minimum energy barrier. All transition state searches are carried out at zero applied pressure.

The BC mechanism, as shown in Fig. 4A, is observed for both reentrant and doubly reentrant geometries. The mechanism proceeds via the liquid-vapor interface sagging toward the system base while pinned to the cap lip. The transition state is observed to occur after the liquid meniscus has contacted the base of the system. This mechanism is highly prevalent across a broad range of structural and chemical properties, such as on nonwetting geometries (see the Supplementary Materials) as well as pillars and nails (*39*, *41*–*44*).

The PC mechanism, illustrated in Fig. 4B, occurs only for the reentrant geometry. The mechanisms proceeds via the liquid-vapor interface impinging under the cap, with the transition state occurring before the interface detaches from the solid surface. Following this, the interface slides down the pillar to contact the base and complete the transition.

Last, the CC mechanism is observed only on the doubly reentrant geometries, as shown in Fig. 4C. Here, the expected PC mechanism is unstable with respect to condensation of liquid inside the cap structure. In this mechanism, the transition begins with the condensation of liquid in one corner of the cap underside, which subsequently grows to fill the cap. The precise location of the transition state can take one of three morphologies, as shown in Fig. 4C (i to iii). Which variant occurs is discussed in the Supplementary Materials. The transition then continues by filling the cap entirely, such that a new free energy minimum is obtained, a suspended state with a liquid-filled cap. An additional energy barrier is required to complete the wetting mechanism: the two separate liquid-vapor interfaces must coalesce by crossing the cap lip before the remainder of the mechanism proceeds exactly as with PC. However, this coalescence barrier is small relative to the condensation barrier presented and decreases further as the lip width is reduced. This mechanism is particularly important to understand as for many applications requiring the doubly reentrant geometry, the liquids are volatile, or else the suspended state needs to be maintained over long time scales. Two such applications are discussed further in subsection Simultaneous Optimization.

Of further note is that the transitions presented here are all MEPs regardless of liquid volatility. The pressure treatment in the free energy functional used in these simulations effectively contacts every point of the system with an external fluid reservoir at constant pressure. Thus, fluids may be exchanged anywhere within the system. For nonvolatile liquids, although the CC condensation mechanism is a minimum energy pathway on the doubly reentrant geometry, CC may not be realized on an experimental time scale. The transition will therefore occur via a noncondensing route, the minimum energy path of which is BC.

#### Minimum energy barriers

Overall, we find that each structure has, at most, two potential transition pathways. Throughout this work, the energy barrier Δ*E*_r_ is expressed relative to the reference energy γ_lv_*B*^2^. The barrier of each pathway is affected differently by the structural parameters, which we summarize in Table 1. In the style of traditional phase diagrams, we present the lowest energy barrier mechanism at each parameter value tested in Fig. 4 (D and E) and so are able to predict the dominant collapse mechanism.

**Table 1:** Geometrical parameters that affect Δ*E*_r_ for each transition mechanism, indicated with filled circles.

	**Reentrant**		**Doubly reentrant**	
	**BC**	**PC**	**BC**	**CC**
*H*_r_	•		•	
*W*_r_	•	•	•	•
*A*_r_		•		•
*L*_r_				•

Beginning with the reentrant geometry, BC and PC compete for the lowest energy collapse mechanism, as shown in Fig. 4D. As shown in Table 1, for all BC transition states, the interface morphology depends on the pillar height *H*_r_ and cap width *W*_r_. As both of these structural parameters increases, the liquid-vapor interface increases in area, leading to an increase in Δ*E*_r_. However, Δ*E*_r_ cannot be increased indefinitely by increasing *H*_r_; as at a critical pillar height, the height-independent PC mechanism becomes the lowest energy transition pathway. As the PC transition state is associated with the liquid wetting the reentrant cap underside, Δ*E*_r_ is increased by expanding the liquid-vapor interfacial area required to do so. This requires *W*_r_ to be maximized and the pillar width *A*_r_ to be minimized. In designing a reentrant structure exhibiting the maximum energy barrier, the mechanistic switch upon increasing *H*_r_ is a key point to highlight as, assuming taller pillars are mechanically weaker than shorter pillars (*27*, *28*), at a given *W*_r_ and *A*_r_, the optimal structure height is that on the BC-PC boundary, reminiscent of the critical pressure case.

Figure 4E shows the lowest energy collapse mechanism for the doubly reentrant geometries, which is markedly different to the (singly) reentrant equivalent. Here, it is the BC and CC mechanisms that compete for the lowest energy path. In Table 1, we find that the minimum energy barrier depends on four parameters: the CC energy barrier depends on *W*_r_, *A*_r_, and the lip depth *L*_r_, whereas the BC barrier depends on *W*_r_ and also *H*_r_. However, ubiquitously, the lowest CC barriers are obtained by minimizing *A*_r_, such that the barrier diagram shown in Fig. 4E is at constant *A*_r_ = 0.05.

Regarding the BC mechanism, the only effect on changing from a reentrant to doubly reentrant geometry is to increase the range of *W*_r_ and *H*_r_ for which the BC mechanism is operative. However, except for the smallest pillar heights, the CC mechanism has a considerably smaller Δ*E*_r_ compared to the BC barrier. This is principally caused by the condensing critical nucleus having a relatively small, energetically unfavorable liquid-vapor interfacial area, compared to the large, energetically favorable solid-liquid interfacial area. Therefore, to maximize the CC barrier, the liquid-vapor interfacial area must be maximized, while minimizing the solid-liquid contact area. This is effectively realized by maximizing *W*_r_ and minimizing *L*_r_ and *A*_r_. As the CC mechanism is independent of the height of the structure, in a similar manner to the reentrant structure, the optimal pillar height is located on the boundary between the two failure mechanisms.

### Simultaneous optimization

Overall, six structural parameters influence the three key wetting properties: *A*_r_ (pillar width), *H*_r_ (pillar height), *L*_r_ (lip depth), *t*_r_ (cap thickness), *W*_r_ (cap width), and the system scale *B*/*B*_ref_ (where *B*_ref_ = 1 μm). Having studied how these parameters affect each individual wetting property, the parameters that antagonistically couple the wetting properties become apparent. First, to reduce the CAH, *W*_r_ must be reduced; but this reduces Δ*P*_c_ and Δ*E*_r_. Second, to increase Δ*P*_c_, the system scale must be reduced; but this reduces Δ*E*_r_. To overcome this unfavorable coupling, we simultaneously optimize the surface structures, which is demonstrated for two example applications: membranes for water purification via membrane distillation, and droplet-based digital microfluidics. To perform this simultaneous optimization, we begin by developing an application-specific scoring function that grades a candidate design against the desired wetting properties. We then optimize the scoring function using two methods. The first is to evaluate the scoring function over the entire parameter range tested, and from this, find the optimum structure. In the second method, we demonstrate that for designs where it is not practical to perform comprehensive wetting property surveys, because of increased surface complexity, for example, genetic algorithms can be used to efficiently perform the simultaneous optimization.

Conventionally in membrane distillation, purification is achieved by passing a heated-contaminated water source over a hydrophobic membrane, through which water vapor is able to pass to collect in a clean water reservoir. However, oils readily foul the membrane, leading to breakthrough of the contaminated liquid into the fresh water reservoir (*2*, *45*). We overcome this using a doubly reentrant structure. To optimize the geometry, we construct a suitable scoring by recognizing that the first priority is for the membrane to be pressure resistant under typical operating conditions: water at 70°C (γ_lv_ = 64.4 mN m^−1^) under pressures of approximately 100 kPa ($\Delta P_{c}^{\text{target}}$) (*46*). To ensure that the suspended state remains stable, the minimum energy barrier must be of the order of 100 k_B_T ($\Delta E_{r}^{\text{target}}$). This is of particular importance for the doubly reentrant geometry to prevent failure via condensation within the texture (*47*). Last, to reduce viscous drag across the membrane, we minimize the CAH and impose the condition that the CAH should not exceed 90° (CAH^cutoff^). The critical pressure, energy barrier, and CAH conditions generate individual scoring functions *S*_P_, *S*_E_, and *S*_C_, respectively

SP=12[1+tanh(ΔPc−ΔPctargetΔPwidth)]SB=12[1+tanh(ΔEr−ΔErtargetΔEwidth)]SC=max(CAHcutoff−CAHCAHcutoff,0) (6)

from which the total score is the geometric mean of these: $\text{Score}={\left(S_{P}S_{B}S_{C}\right)}^{\frac{1}{3}}$. For *S*_P_ and *S*_B_, a tanh profile is selected to appropriately localize critical pressures and energy barriers within a range of suitable operating conditions. This leads to the widths chosen here as Δ*P*_width_ = 0.5 and Δ*E*_width_ = 5 × 10^−5^. Meanwhile, the linear function for *S*_C_ aims to ensure that low-CAH structures are always favored.

By maximizing this six-dimensional scoring function using either the results from the wetting property survey or a genetic algorithm, the optimal structure is obtained with a score of 0.794. The optimal parameters are (*A*_r_, *H*_r_, *L*_r_, *t*_r_, *W*_r_, *B*/*B*_ref_) = (0.05, ≥0.17, 0.17, 0.05, 0.27, 0.32). The optimum system scale of 320 nm is notably similar to that of springtail cuticles (*9*). Both the springtail cuticle and membrane design have been selected for pressure-resistant liquid shedding ability, while allowing the unimpeded movement of gasses through the surface. The membrane design proposed here may therefore reflect a natural optimum for robust gaseous diffusion. The optimum design yields the properties: Δ*P*_c_ = 162 kPa, Δ*E* = 1.25 × 10^3^ k_B_T, and CAH = 42° (θ_a_ = 165°, θ_r_ = 123°). This CAH is typical of currently manufactured reentrant microtextures [see, for example, (*27*, *29*, *31*)].

By studying the individual wetting properties, we can rationalize the optimal structural design. The optimal value of *A*_r_ represents the minimum pillar width tested, whose sole function is to maximize Δ*E*_r_. *H*_r_ reflects the observation that the maximum critical pressure is achieved at *H*_r_ ≥ *H*_c_. *L*_r_ + *t*_r_ optimizes the CAH, while the specific value of *L*_r_ maximizes Δ*E*_r_. Last, the small value of *W*_r_ reduces the CAH while retaining a high Δ*P*_c_ due to the small system scale.

**Fig. 5:**
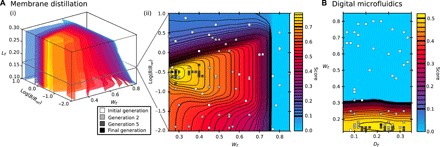
Simultaneous optimization of the three wetting properties for membrane distillation and digital microfluidics applications. (**A**) (i) 3D contour plot of the membrane distillation scoring function at fixed *H*_r_ = 0.3, *A*_r_ = 0.05, and *t*_r_ = 0.05. Each surface is a surface of constant score. (ii) A 2D slice of the 3D contour plot at the optimal *L*_r_ = 0.17. Square data points show the initial (white), second (light gray), fifth (dark gray), and final (black) generations of the genetic algorithm, projected onto the 2D plane. (**B**) Scoring function for the digital microfluidics application, projected onto the *H*_r_ = 0.3 plane at fixed *B* = 100 μm, also showing the successive generations of the genetic algorithm population.

The scoring function at fixed optimal values of *A*_r_, *H*_r_, and *t*_r_ is shown in Fig. 5A (i) as a 3D contour plot. A 2D cut through this is shown in Fig. 5A (ii) at the optimal *L*_r_, to show that the optimal structure scale is bounded by the critical pressure criterion from above and the minimum energy barrier criterion from below. Also shown in Fig. 5A (ii) are projections of successive generations of the genetic algorithm. The optimal structure was located after 20 generations, requiring the sampling of only 0.01% of the 7.2 × 10^6^ possible structures considered overall.

The four examples of manufactured doubly reentrant surfaces feature system sizes of 1 to 100 μm, as smaller scales are currently challenging to manufacture (*13*–*16*). We now choose to optimize a structure whose manufacture has already been demonstrated, so we fix *B* = 100 μm in accordance with the texture designed in a recent work by Liu and Kim (*16*). A leading-edge application for this is surfaces designed for digital microfluidics (*8*, *21*). Devices fail when droplets become immobilized by pinning to the surface, particularly problematic for low–surface tension solvents (*21*) or for reactive processes where the surface tension is variable and hard to predict.

Both of these problems are readily overcome using the doubly reentrant geometry. We demonstrate this by optimizing a surface structure for use within a particularly challenging scenario—digital microfluidics using microliter volume droplets of *n*-hexane (γ_lv_ = 27.4 mN m^−1^). The pressure within such a droplet (88 Pa) introduces the target pressure for use in the scoring scheme Eq. 6: $\Delta P_{c}^{\text{target}}$ = 100 Pa, with the width Δ*P*_width_ = 0.005. Furthermore, as the CAH should be minimized, we impose the condition CAH^cutoff^ = 50°. At the imposed length scale used, the barrier score *S*_B_ ≈ 1 (Δ*E* is of the order of 10 × 10^10^ k_B_T), meaning that we choose to optimize the score (*S*_P_*S*_C_)^1/2^.

Overall, the optimum structure, 0.508, is obtained using both the wetting property survey and the genetic algorithm. The optimum parameters are (*H*_r_, *D*_r_, and *W*_r_) = (≥0.13, 0.22, and 0.16), yielding the properties: Δ*P*_c_ = 105 Pa, CAH = 25°. The pillar width *A*_r_ and ratio of *L*_r_ to *t*_r_ become free parameters to choose. A 2D contour plot of the scoring function at constant *H*_r_ = 0.3 is shown in Fig. 5B, in which projections of successive generations of the genetic algorithm are shown. Here, the algorithm converged after 14 generations, requiring 2.2% of the entire population to be sampled.

For both the membrane distillation and digital microfluidics applications, the sensitivity of the optimized structural dimensions and properties can be assessed relative to the choice of scoring function parameters. This is achieved through reoptimizing the geometries when each parameter in the scoring functions shown in Eq. 6 were varied individually by ±5%. It is found that the optimized membrane distillation geometry is insensitive to the parameter variation. This observation also applies to the optimized digital microfluidics geometry, except in the scenario where CAH^cutoff^ is reduced by 5%. In this case, the optimal CAH is reduced by 13%, and the optimal critical pressure is reduced by 17%. However, this variation is due to the CAH^cutoff^ reduction (2.5°) being relatively large compared to the low optimal CAH (25°) for this application.

The manufactured structure reported by Liu and Kim (*16*) is similar to the optimum geometry. However, the key difference is that the optimal geometry has a considerably shorter pillar height *H*_r_ than the manufactured geometry by a factor of 3.7 times. This is due to the unexpectedly short critical height required, as discussed in the critical pressure section.

## CONCLUSIONS AND OUTLOOK

Overall, to optimize the wetting properties of the reentrant and doubly reentrant surface texture for a vast variety of potential applications, we began by developing three computational strategies to comprehensively survey the key surface wetting properties: the CAH, critical pressure, and minimum energy barrier to the wetting transition. This was achieved for both wetting and nonwetting liquids (shown in the Supplementary Materials).

In the CAH study, we identified four major receding mechanisms, of which only two had previously been reported, and defined the structural dimensions where each is operative. For all receding mechanisms, we were able to develop and analyze quantitative models that were robustly validated against our simulation results.

In the critical pressure study, three failure mechanisms were observed and quantified as a function of the structural parameters. However, upon comparison with the simulation data, the prevailing and widely used critical pressure models were found to be considerably oversimplified. This leads to a particularly poor description of the liquid-vapor interface morphology, meaning that manufactured structures are many times taller (and mechanically weaker) than necessary. By developing a more sophisticated model, we were able to achieve both quantitative accuracy of the critical pressures and success at modeling the complex interface morphologies as capillary bridges.

In the minimum energy barrier study, we identified three failure mechanisms, quantified each barrier across the structural parameter space, and assessed which mechanism was most likely for a given geometry. Crucially, we not only showed how the doubly reentrant geometry is prone to condensation within the cap but also deduced effective designs to mitigate against this.

Last, we found the structural features that tend to maximize the critical pressure, minimize the energy barrier, and maximize the CAH. As it was not possible to optimize a surface geometry with respect to each individual wetting parameter, we performed the optimization by considering all three simultaneously. This was achieved in two ways for the optimum design of both membranes for water purification and surfaces for digital microfluidics. First, using the comprehensive wetting property surveys, we were able to evaluate and locate the maximum of a combined scoring function. However, we then demonstrated that a genetic algorithm was able to efficiently locate the optimum design in the six-dimensional parameter space. Although the designs tested here featured a relatively small number structural degrees of freedom, going forward, we highlight such optimization techniques as being powerful tools in designing more complex structures for special wettability applications. The computational techniques developed here are highly versatile and can be used for any mesoscopically structured surface in contact with multiple fluid phases.

Coupled with recent, substantial developments in fabrication techniques (including 3D printing, fluidization of polymer micropillars, and lithographic methods), we believe that the multifaceted optimization strategy presented here will be a powerful approach to designing real-world superomniphobic surfaces. In the future, an additional step will be to consider the mechanical reliability and scalability of manufactured designs, in which we have contributed to this discussion with our large reduction in the necessary pillar height.

## METHODS

### Diffuse interface model

The simulations used to compute the CAH, critical pressure, and minimum energy barrier all used the same diffuse interface model and system discretization. Specific system setups for each wetting property are presented in the Supplementary Materials. Within the bifluidic diffuse interface model used, the order parameter ϕ(**r**) was chosen to represent the local composition at point **r** (ϕ = 1 in the pure liquid phase, and ϕ = −1 in the pure vapor phase at zero applied pressure). On the basis of a previous work, the free energy functional Ψ[ϕ] is composed of three terms (*41*)

$$\Psi [\phi ]=\Psi_{i}+\Psi_{s}-\Delta {\mathrm{PV}}_{l}$$ (7)

Ψ*_i_* is the isotropic free energy term, expressed as an integral over the entire system volume *V*

$$\Psi_{i}=\int_{V}(\frac{1}{\epsilon }(\frac{1}{4}\phi^{4}-\frac{1}{2}\phi^{2}+\frac{1}{4})+\frac{\epsilon }{2}{|\nabla \phi |}^{2})\mathrm{dV}$$ (8)

enforcing the equilibrium values of ϕ = ±1 via the double well potential, and exacting an energetic penalty for forming an interface of width ϵ. This leads the liquid-vapor surface tension $\gamma_{\text{lv}}=\sqrt{8/9}$.

Ψ*_i_* is the fluid-solid interaction term, expressed as an integral over the surface area

$$\Psi_{s}=\int_{S}h(-\frac{1}{6}\phi_{s}^{3}+\frac{1}{2}\phi_{s}+\frac{1}{3})\mathrm{dS}$$ (9)

where ϕ*_s_* is the value of ϕ at the surface. *h* is the wetting parameter and is related to the microscopic contact angle θ_°_ through $h=-\sqrt{2}\text{cos}\theta_{{}^{\circ}}$. The cubic wetting potential negates spurious compositional changes close to the surface by ensuring that ϕ*_s_* is equal to the bulk composition (*48*).

Within the external pressure term, the total liquid volume is calculated from

$$V_{l}=\int_{V}\frac{\phi +1}{2}\mathrm{dV}$$ (10)

The simulation system is discretized into a cubic lattice of *N_x_* × *N_y_* × *N_z_* nodes, in which each node is either located within the solid structure, on the solid surface, or in the fluid bulk.

### Genetic algorithm

We began by randomly sampling the parameter space to generate an initial population of 40 surface structures. These were ranked on the basis of score, and the top 20 were retained for breeding. Candidate pairs for breeding were selected at random, and breeding occurred if the geometric mean of their scores was greater than a random number between 0 and 1. The offspring were equally likely to inherit each attribute from either parent. Each attribute was then mutated if a random number was less than the current mutation probability *p_i_* (set initially at 0.5). For the discrete structural variables, a mutation changed the attribute randomly by between −3 and 3 lattice spacings. For the continuous variable (system scale), the change in log(*B*/*B*_ref_) was selected randomly from the range −0.3 to 0.3. The mutation probability was reduced each generation, such that *p*_*i*+1_ = *p*_0_× (SD of scores in previous generation)^1/2^. Any offspring bred or mutated outside the testable parameter range was mutated back into the testable parameter range. The algorithm was deemed to have converged when the mutation rate decreased to zero.

## Supplementary Material

http://advances.sciencemag.org/cgi/content/full/5/6/eaav7328/DC1

Download PDF

Movie S1

Movie S2

Movie S3

Movie S4

## SUPPLEMENTARY MATERIALS

Supplementary material for this article is available at http://advances.sciencemag.org/cgi/content/full/5/6/eaav7328/DC1 Section S1. Supplementary Materials and Methods Section S2. Extended discussion for θ_°_ = 60° Section S3. Results and discussions for θ_°_ = 110° Fig. S1. CAH simulation setups. Fig. S2. Decomposition of CAH into advancing and receding contact angles. Fig. S3. Analytic models for the receding mechanisms. Fig. S4. Illustrated critical pressure model parameters. Fig. S5. Analysis and comparison of the circular arc and nodoid models of the sagging depth of the liquid-vapor interface at the critical pressure. Fig. S6. Effects of the reentrant and doubly reentrant structural parameters on each transition type. Fig. S7. Extended analysis of the lowest energy reentrant and doubly reentrant transition mechanisms. Fig. S8. CAH mechanisms for θ_°_ = 110°. Fig. S9. Critical pressure analysis for θ_°_ = 110°. Fig. S10. Individual and lowest energy transition mechanisms for θ_°_ = 110°. Movie S1. BC mechanism for a doubly reentrant geometry at θ_°_ = 60°. Movie S2. PC mechanism for a reentrant geometry at θ_°_ = 60°. Movie S3. CC mechanism for a doubly reentrant geometry at θ_°_ = 60°. Movie S4. PC mechanism for a reentrant geometry at θ_°_ = 110°. References (*49*–*54*)
