# Supplementary material for: Multifaceted design optimization for superomniphobic surfaces
Source: Sci Adv. 2019 Jun 21;5(6):eaav7328. doi: 10.1126/sciadv.aav7328 (PMC6719413; doi:10.1126/sciadv.aav7328)
Supplement: http://advances.sciencemag.org/cgi/content/full/5/6/eaav7328/DC1 [file supp_5_6_eaav7328__index.html]

Science Advances | Science Advances

## Supplementary Materials

**The PDF file includes:**

- Section S1. Supplementary Materials and Methods
- Section S2. Extended discussion for θ° = 60°
- Section S3. Results and discussions for θ° = 110°
- Fig. S1. CAH simulation setups.
- Fig. S2. Decomposition of CAH into advancing and receding contact angles.
- Fig. S3. Analytic models for the receding mechanisms.
- Fig. S4. Illustrated critical pressure model parameters.
- Fig. S5. Analysis and comparison of the circular arc and nodoid models of the sagging depth of the liquid-vapor interface at the critical pressure.
- Fig. S6. Effects of the reentrant and doubly reentrant structural parameters on each transition type.
- Fig. S7. Extended analysis of the lowest energy reentrant and doubly reentrant transition mechanisms.
- Fig. S8. CAH mechanisms for θ° = 110°.
- Fig. S9. Critical pressure analysis for θ° = 110°.
- Fig. S10. Individual and lowest energy transition mechanisms for θ° = 110°.
- Legends for movies S1 to S4
- References (*49*–*54*)

Download PDF

**Other Supplementary Material for this manuscript includes the following:**

- Movie S1 (.mp4 format). BC mechanism for a doubly reentrant geometry at θ° = 60°.
- Movie S2 (.mp4 format). PC mechanism for a reentrant geometry at θ° = 60°.
- Movie S3 (.mp4 format). CC mechanism for a doubly reentrant geometry at θ° = 60°.
- Movie S4 (.mp4 format). PC mechanism for a reentrant geometry at θ° = 110°.

**Files in this Data Supplement:**

- Adobe PDF - aav7328\_SM.pdf
