# Supplementary material for: Multifaceted design optimization for superomniphobic surfaces
Source: Sci Adv. 2019 Jun 21;5(6):eaav7328. doi: 10.1126/sciadv.aav7328 (PMC6719413; doi:10.1126/sciadv.aav7328)
Supplement: Download PDF [file aav7328_SM.pdf]

Science Advances
AAAS

advances.sciencemag.org/cgi/content/full/5/6/eaav7328/DC1

Supplementary Materials for

Multifaceted design optimization for superomniphobic surfaces

J. R. Panter, Y. Gizaw, H. Kusumaatmaja*

*Corresponding author. Email: halim.kusumaatmaja@durham.ac.uk

Published 21 June 2019, *Sci. Adv.* **5**, eaav7328 (2019)
DOI: 10.1126/sciadv.aav7328

The PDF file includes:

Section S1. Supplementary Materials and Methods
Section S2. Extended discussion for $\theta_o = 60°$
Section S3. Results and discussions for $\theta_o = 110°$
Fig. S1. CAH simulation setups.
Fig. S2. Decomposition of CAH into advancing and receding contact angles.
Fig. S3. Analytic models for the receding mechanisms.
Fig. S4. Illustrated critical pressure model parameters.
Fig. S5. Analysis and comparison of the circular arc and nodoid models of the sagging depth of the liquid-vapor interface at the critical pressure.
Fig. S6. Effects of the reentrant and doubly reentrant structural parameters on each transition type.
Fig. S7. Extended analysis of the lowest energy reentrant and doubly reentrant transition mechanisms.
Fig. S8. CAH mechanisms for $\theta_o = 110°$.
Fig. S9. Critical pressure analysis for $\theta_o = 110°$.
Fig. S10. Individual and lowest energy transition mechanisms for $\theta_o = 110°$.
Legends for movies S1 to S4
References (*49–54*)

Other Supplementary Material for this manuscript includes the following:

(available at advances.sciencemag.org/cgi/content/full/5/6/eaav7328/DC1)

Movie S1 (.mp4 format). BC mechanism for a doubly reentrant geometry at $\theta_o = 60°$.
Movie S2 (.mp4 format). PC mechanism for a reentrant geometry at $\theta_o = 60°$.
Movie S3 (.mp4 format). CC mechanism for a doubly reentrant geometry at $\theta_o = 60°$.
Movie S4 (.mp4 format). PC mechanism for a reentrant geometry at $\theta_o = 110°$.

Section S1. Supplementary Materials and Methods

1.1 Contact angle hysteresis

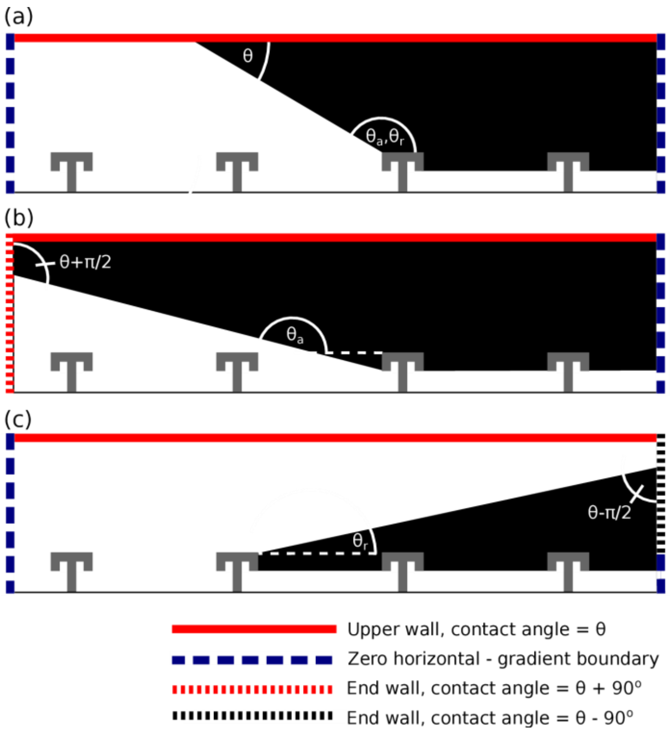

Fig. S1: CAH simulation setups. Microchannel boundary conditions for: (a) the conventional setup, (b) the advancing case where $\theta < 20°$, (c) the receding case where $\theta > 160°$.

To simulate the advancing and receding scenarios, we adapted the microchannel setup used by Mognetti and Yeomans (*26*). In this, a single column of surface structures patterned the base of the microchannel with microscopic contact angle $\theta_{\circ} = 60°$. The base of each individual structure was centred within a square of $N_x \times N_y = 60 \times 60$ lattice spacings, where each lattice spacing is equal to the interface width ϵ. Thus, in units of ϵ the system size $B = 60$. The top of the channel was capped with a smooth surface of variable contact angle θ, and was set above the top of the surface structures at a height of 40 lattice units. This height was chosen to ensure that the top surface did not influence the advancing and receding interface morphologies, which is evidenced as the interface being planar at the top surfaces. For the majority of CAH simulations, the boundary conditions employed were as shown in fig. S1(a). Here, the gradient in ϕ perpendicular to the end walls was fixed at zero, enforcing bulk fluid behaviour. To determine θ_a, an iterative process was used in which θ was incremented and the free energy minimised via the L-BFGS algorithm (*49*, *50*), up to the point at which the advancing interface morphology was obtained to a precision of 0.1°. At convergence, the advancing contact angle θ_a = 180°- θ. An identical process was used to determine the receding interface morphology. At convergence, the receding contact angle θ_r = 180°- θ. The relationships between θ_a, θ_r, and θ are indicated in fig. S1(a).

Special modifications to the microchannel were required when advancing at $\theta < 20°$, or receding at $\theta > 160°$, as the diffuse interface caused a reduction in contact angle accuracy. In the former case, as shown in fig. S1(b), the advancing-end boundary was replaced by a wall with contact angle equal to θ + 90°. As before, θ_a = 180°- θ. In the latter case, as shown in fig. S1(c), the receding-end boundary was partially replaced by a wall with contact angle equal to θ - 90°. As before, θ_r = 180°- θ. The height at which this wall started was between the lip bottom and top, so as not to artificially affect the interface morphology, or receding contact angle. In both cases, the end walls were located sufficiently far from the contact line pinning location that the interface was planar in the end wall vicinity.

1.2 Critical pressure

For each critical pressure simulation, a double-resolution system was employed in which the solid structure was centred within a simulation volume of $N_x \times N_y \times N_z = 240\times240\times(H + D + 16)$ where the lattice spacing was equal to $\epsilon/2$. Thus, in units of ϵ, the system size $B = 120$. This was used to ensure that the contact line pinning at the critical pressure was not sensitive to the diffuse interface width. As the critical pressure interface morphologies never broke the square symmetry of the system, computational efficiency was increased by simulating one quarter of the system: $x \in [0,N_x/2], y \in [0,N_y/2], z \in [0,N_z]$. Mirror boundary conditions at $x = 0, N_x/2$ and $y = 0, N_y/2$ maintained the square symmetry. The z-gradient at $z = N_z$ was set to zero to enforce bulk fluid behaviour. Each system was initiated in a zero-pressure suspended state, then an iterative process was used to find the critical pressure by an

iterative process of pressure increase and energy minimisation. The largest ΔP_r at which the suspended state remained stable was determined with a precision of $0.01\gamma_{lv}/B$.

1.3 Minimum energy barriers

Here, we develop a rapid and precise transition state search method, suitable for any surface design, which can be readily implemented in pre-existing minimisation routines.

The gradient-squared method employed to survey the transition states shares similarities to that used recently for atomistic simulations (*51*). The free energy minima are found by locating the stationary points of Ψ at which all eigenvalues are positive. If

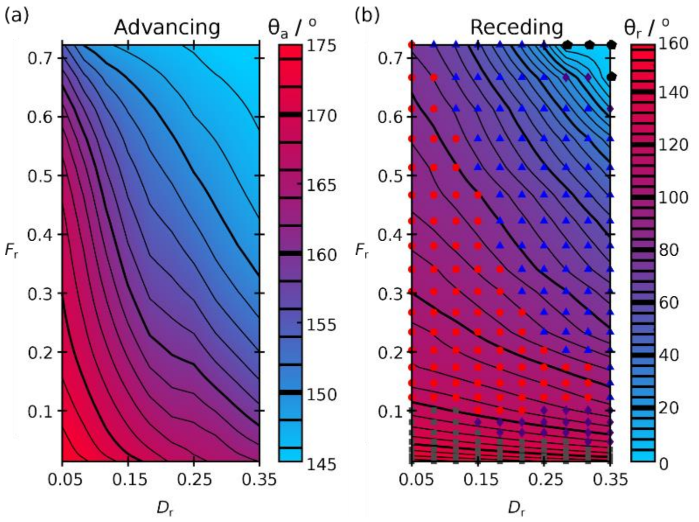

Fig. S2. Decomposition of CAH into advancing and receding contact angles. Contour plots of the individual advancing (a) and receding (b) contact angles, as the total lip depth D_r and area fraction F_r are varied. For the receding case, the receding mechanisms are indicated: bridge depinning (grey squares), edge depinning (red circles), lip depinning (blue triangles), non-depinning (black pentagons), and hybrid-depinning (purple diamonds).

we transform the landscape such that we minimise the gradient-squared, Γ,

$$\Gamma = \sum_{ijk} \left(\frac{\partial \Psi}{\partial \phi_{\text{ijk}}} \right)^2 , \tag{1}$$

then all stationary points become minima of Γ, with $\Gamma = 0$. Inflection points also become minima of Γ, but have $\Gamma \neq 0$. Since the transformed landscape exhibits a large number of basins of attraction, care must be taken to initialise the system close to a transition state. This was achieved through using the Doubly Nudged Elastic Band algorithm (*40*) once for each transition type to locate an approximate transition state. An effective method was to use these as system initialisations, and by making small changes to the surface structure, we could ensure that we never escaped the basin of attraction of the transition state (where it existed), and so reliably converged on the transition state upon minimising Γ.

Each transition state was obtained on a single structural replica within a cubic simulation volume of $N_x \times N_y \times N_z = 60 \times 60 \times 60$ where the spacing was equal to ϵ. Thus, the system size $B = 60$. We limited the range of geometries tested as we imposed a minimum $C_r(min) = 0.1$ to ensure the diffuse interface did not dominate the wetting behaviour, thus $A_r < W_r - 2C_r(min)$.

Section S2. Extended discussion for θ_o = 60°

2.1 Advancing and receding contact angles

In fig. S2, we show the advancing and receding contact angles measured from simulations separately. For all practical surfaces used in superomniphobic applications, the advancing contact angle θ_a exceeds 160°, shown in fig. S2(a). θ_a < 160° is only observed for large lip depths and area fractions, both of which are effective at pinning the advancing interface to the advancing bottom edge of the cap. Although limited, a direct comparison with previous experimental results can be made at $F \approx 0.196$, D_r = 0.087 and 0.17, in which octane was found to make a microscopic contact angle with surface of 60° (*27*). Experimentally, $\theta_a \approx$ 157° and 152° for each D_r respectively, compared to θ_a = 169° and 163° measured in this simulation. The 7% difference between each value is negligible compared to the experimental precision of measuring such large contact angles.

The receding contact angles shown in fig. S2(b) span almost the entire possible contact angle range. We highlight the small variation in θ_r with D_r at small area fractions F_r, compared to the large variation in θ_a. It is these contrasting variations which are responsible for the optimal values of D_r (or L_r) due to the CAH, presented in the simultaneous optimisation. We now compare the simulated θ_r with the same experiments as discussed for θ_a (*27*). At D_r = 0.087 and 0.17, experimentally $\theta_r \approx$ 108° and 99° respectively. Via simulation, we observe θ_r = 108° and 103° respectively. Within the error margins (±2° in experiment, ±1° in simulation), the experimental and simulation results are in very good agreement.

2.2 Receding models

2.2.1 Bridge depinning

In order to model the bridge depinning mechanism, we begin by considering the maximum pinning force a capillary bridge can achieve when being strained parallel to it's axis, illustrated in fig. S3(a)(i). In this, we show a 2D slice through the centre of an axisymmetric system. The axis of symmetry is denoted by the vertical dotted line. Assuming the contact angle at the three phase contact line is uniform throughout, at the point of depinning the maximum pinning force **f** is expressed as

$$|\boldsymbol{f}| = \gamma_{\mathrm{lv}} m \cos \theta_{\circ}^{\max}, \tag{2}$$

where m is the contact line length, and $\theta_\circ^{max}$ is the corrected contact angle. $\theta_\circ^{max}$ is defined as $\theta_\circ^{max}$ = max[$\theta_\circ$,$\pi/2$].

In receding systems, the capillary bridge is strained along the receding direction, shown in figs. S3(a)(ii,iii). Following a previous derivation (*20*), but augmented with our treatment of $\theta_\circ^{max}$, two effects arise. Firstly the direction of the pinning force **f** balances the capillary forces arising from the front and receding edge of the cap, and so forms an angle which bisects the receding contact angle (*30*), shown in fig. S3(a)(ii). Secondly, the contact angle around the contact line varies between a maximum value at the innermost edge (right hand side of fig. S3(a)(iii)), to a minimum value at the outermost edge (left hand side of fig. S3(a)(iii)). At the innermost edge, the receding angle increases the local contact angle to $\theta_\circ^{max} + \pi - \theta_r$. At the outermost edge, the contact angle cannot be reduced from $\theta_\circ^{max}$, otherwise the contact line would depin. The pinning force **f** is assumed to depend on the average contact angle θ, approximated as $\bar{\theta} = \theta_\circ^{\max} + (\pi - \theta_r)/2$.

At equilibrium, the horizontal component of the microscopic pinning force $\mathbf{f}_h$ must balance the macroscopic receding force **F**, leading to

$$\begin{aligned} |\boldsymbol{f}_{\mathrm{h}}| &= \gamma_{\mathrm{lv}} m \sin \bar{\theta} \sin\left(\frac{\pi}{2} - \frac{\theta_{\mathrm{r}}}{2}\right), \\ &= \gamma_{\mathrm{lv}} m \cos\left(\theta_{\circ}^{\max} - \frac{\theta_{\mathrm{r}}}{2}\right) \cos \frac{\theta_{\mathrm{r}}}{2}. \end{aligned} \tag{3}$$

On the macroscale, the force **F** required to displace a contact line of length B is expressed via the Young-Dupré equation for the work of adhesion: $|\mathbf{F}| = \gamma_{lv} B(1 + \cos\theta_r)$. Thus, by equating $|\mathbf{f}_h| = |\mathbf{F}|$ and manipulating the equality, we arrive at a general expression for the receding contact angle for liquids of all wettabilities:

$$\tan \frac{\theta_{\mathrm{r}}}{2} = \frac{2B}{m \sin \theta_{\circ}^{\max}} - \frac{1}{\tan \theta_{\circ}^{\max}}. \tag{4}$$

In the case of wetting liquids, for which $\theta_\circ^{max} = \pi/2$, this simplifies to

$$\tan \frac{\theta_{\mathrm{r}}}{2} = \frac{2}{4 W_{\mathrm{r}} \alpha}, \tag{5}$$

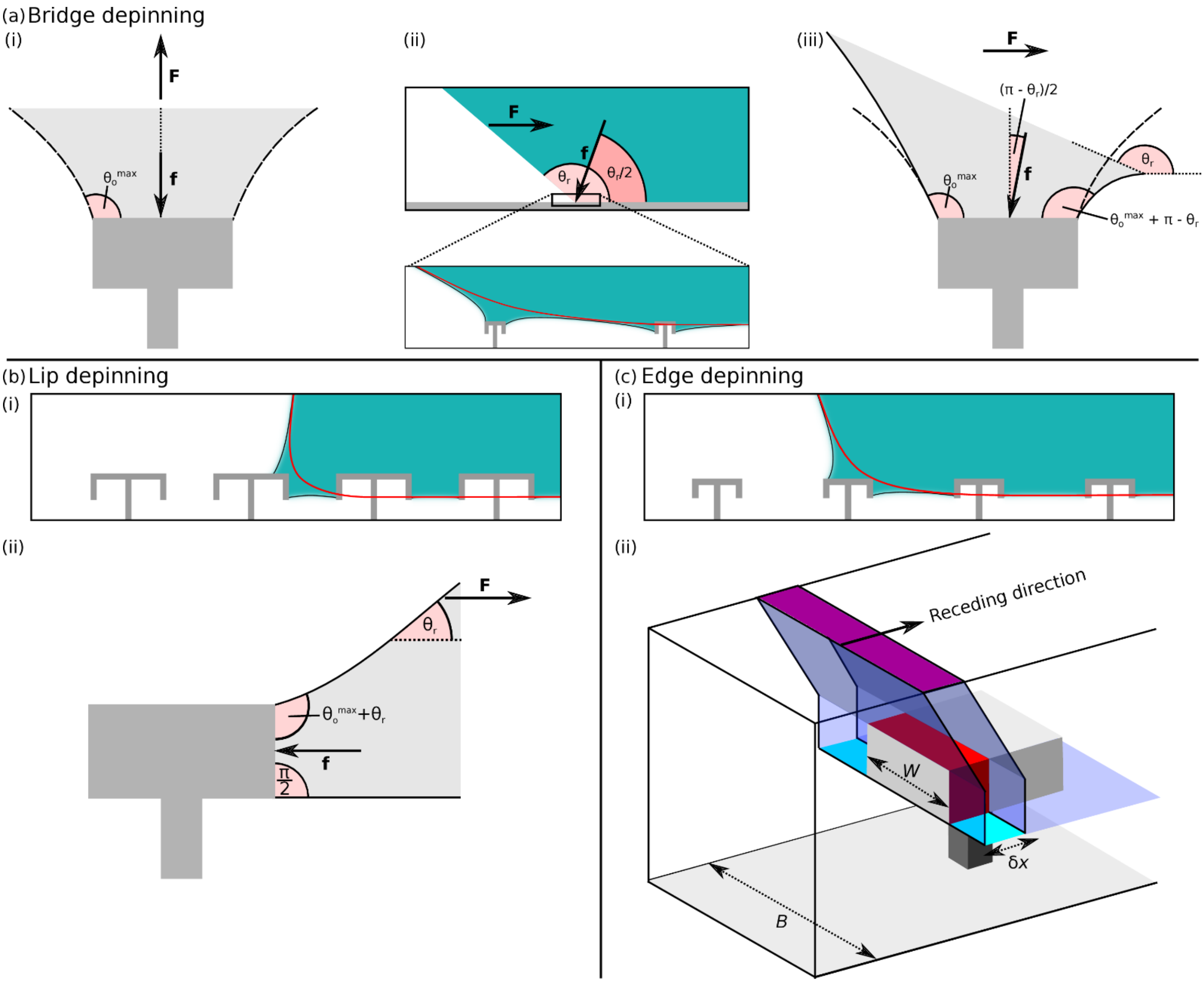

Fig. S3. Analytic models for the receding mechanisms. (a) Bridge depinning model. (i) Force balance occurring at the point of depinning for a vertically strained capillary bridge. The solid reentrant geometry is shown in dark grey, the liquid in pale grey. The system is axisymmetric about the vertical dotted line. (ii) Macroscopic view of the receding interface (top), with magnification about the three-phase contact line to observe the bridge depinning mechanism (bottom). (iii) Model of a single capillary bridge being strained in the receding direction. Dashed black lines indicate the original liquid-vapour interface shape for the vertically strained bridge in (i), whilst the solid black lines indicate the strained interface shape. (b) Lip depinning model. (i) A 2D slice along the centre of the microchannel showing the lip depinning mechanism, in which the liquid (blue) is pinned to the side of the cap. (ii) Model of the lip depinning mechanism in which the liquid meniscus (pale grey) is strained parallel to the receding direction. (c) Edge depinning model. (i) A 2D slice along the centre of the microchannel showing the edge depinning mechanism. (ii) Model of the edge depinning mechanism in which the liquid vapour interface (translucent blue) is displaced by a distance δx along the length of the microchannel. The areas highlighted in purple, red, and cyan, indicate the change in liquid contact area with the top surface, cap, and vapour phase respectively.

where we have substituted the reduced perimeter m/B for that appropriate for square caps: $m/B = 4W_r\alpha$.

2.2.2 Lip depinning

In the lip depinning scheme, the geometry of the capillary-bridge depinning mechanism is markedly altered, shown in fig. S3(b). At zero applied pressure, the liquid-vapour interface pinned to the bottom of the caps is planar, making a contact angle of $\pi/2$ with the cap side. Thus, the microscopic pinning force **f** acts anti-parallel to the macroscopic receding force **F**, as shown in fig. S3(b)(ii). The effect of **F** is therefore to change the contact angle around the contact line, varying from a maximum value ($\theta_\circ^{max} + \theta_r$) at the top of the cap, to a minimum value ($\pi/2$) at the bottom of the cap. We make the assumption that **f** depends on the average contact angle $\bar{\theta} = \frac{\theta_\circ^{\max}}{2} + \frac{\theta_r}{2} + \frac{\pi}{2}$, leading to

$$|\boldsymbol{f}| = \gamma_{\mathrm{lv}} m \sin\left(\frac{\theta_\circ^{\max}}{2} + \frac{\theta_{\mathrm{r}}}{2} + \frac{\pi}{2}\right). \quad (6)$$

For wetting liquids with $\theta_\circ^{\max} = \frac{\pi}{2}$, this simplifies to

$$|\boldsymbol{f}| = \gamma_{\mathrm{lv}} m \cos\frac{\theta_{\mathrm{r}}}{2}. \quad (7)$$

Finally, we equate |**f**| and |**F**| to yield an expression for the receding contact angle due to lip depinning,

$$\cos\frac{\theta_{\mathrm{r}}}{2} = \frac{2(W_{\mathrm{r}} + D_{\mathrm{r}})\alpha}{2}, \quad (8)$$

where we have substituted the reduced perimeter m/B for that appropriate for rectangular caps sides: $m/B = 2(W_r + D_r)\alpha$. We note that for non-wetting surfaces ($\theta_\circ > \pi/2$), as the liquid-vapour interface does not pin to the cap underside in the suspended state, the lip depinning mechanism cannot occur.

2.2.3 Edge depinning

The final model we consider is that of edge depinning for wetting liquids, illustrated in fig. S3(c). We approximate the mechanism as both the liquid-vapour interface and three-phase contact line sliding without changing shape. Thus, we are able to evaluate the energy change for this process over a small displacement δx of the contact line along the microchannel. The energy change is expressed as the sum of contributions arising from sliding across the top surface of the microchannel, sliding across the surface of the cap, and reducing the liquid-vapour interfacial area, such that

$$\begin{aligned}\delta E \; &= \gamma_{\mathrm{sv}}^{\mathrm{top}} B\delta x - \gamma_{\mathrm{sl}}^{\mathrm{top}} B\delta x \\ &+ \gamma_{\mathrm{sv}}^{\mathrm{cap}}(W + 2D)\delta x - \gamma_{\mathrm{sl}}^{\mathrm{cap}}(W + 2D)\delta x \\ &- \gamma_{\mathrm{lv}}(B - W)\delta x. \end{aligned} \quad (9)$$

This is readily simplified using the Young equation to yield an expression for the force required to exact the displacement of the contact line,

$$\frac{\delta E}{\delta x}\gamma_{\mathrm{lv}}\left[B\cos\theta_{\mathrm{top}} + (W + 2D)\cos\theta_\circ - (B - W)\right]. \quad (10)$$

At the point of receding, this force is equal to zero. By realising that $\theta_r = \pi - \theta_{\mathrm{top}}$, this yields

$$\cos\theta_{\mathrm{r}} = (W_{\mathrm{r}} + 2D_{\mathrm{r}})\cos\theta_\circ + W_{\mathrm{r}} - 1. \quad (11)$$

2.3 Critical pressure models

2.3.1 Critical pressure derivation

To model the critical pressure, we improve on the derivation presented by Tuteja *et al.* (*17*, *29*) to capture the actual contact line morphology. We begin by considering a typical system at the critical pressure, of size B, cap width W, illustrated in fig. S4. At the critical pressure, the pinning force of the contact line balances the force due to the pressure, such that

$$(\Delta P_{\mathrm{c}}\gamma_{\mathrm{lv}}/B)(B^2 - S) = \gamma_{\mathrm{lv}} m \sin\theta_\circ^{\min}, \quad (12)$$

where ΔP_c is the reduced critical pressure (normalised with respect to γ_{lv}/B). $(B^2 - S)$ is the projected area occupied by the liquid phase, shown in fig. S4(b). As in the contact angle hysteresis study, m is the contact line perimeter, and again we take care to correctly capture the true pinning force by using the corrected contact angle, where $\theta_\circ^{min} = \min[\theta_\circ, \pi/2]$ if the contact line is pinned to the outer cap edge, or $\theta_\circ^{min} = \pi/2$ if the contact line is pinned to the inner cap edge. We note that the former case is appropriate for wetting on (singly) reentrant geometries, or doubly reentrant geometries where $\theta_\circ > \pi/2$. The latter case is appropriate for doubly reentrant geometries where $\theta_\circ < \pi/2$. In all cases, we make the assumption that the average contact angle is equal to $\theta_\circ^{min}$.

To model the contact line perimeter, we assume that the pinned width of the contact line is W', which is different from the cap width W by a distance a. For pinning on the inner lip (for wetting liquids on doubly reentrant structures as shown in fig. S4), we

expect a to be approximately twice the lip width l_r. For pinning on the outer lip (for reentrant geometries, or non-wetting liquids on doubly reentrant geometries), we expect $a \approx 0$. Overall, $m = 4\alpha W'$, where the shape parameter α is used to smoothly vary the possible perimeter shapes from circular ($\alpha = \pi/4$) to square ($\alpha = 1$). To model the projected vapour-occupied area S, we again use the corrected width W' and shape parameter α to model the contact line shape, yielding $S = \alpha W'^2$. The critical pressure model is obtained by manipulating Eq. (12),

$$\begin{aligned}\Delta P_c &= B \sin\theta_\circ^{\min} \frac{m}{B^2 - S},\\ &= \sin\theta_\circ^{\min} \frac{4\alpha W' B}{B^2 - \alpha W'^2},\\ &= \sin\theta_\circ^{\min} \frac{4\alpha}{\frac{1}{W_r'} - \alpha W_r'},\end{aligned} \tag{13}$$

where W_r' is the reduced corrected width W'/B.

2.3.2 Critical height derivation

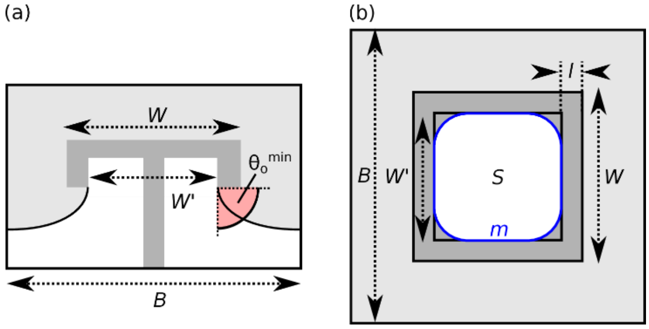

Fig. S4. Illustrated critical pressure model parameters. (a) Side view of a 3D system at the critical pressure. In this, the liquid (pale grey) sags beneath the cap (dark grey),and the contact line is pinned with contact angle $\theta_\circ^{min}$. (b) Underside view of the same system, showing the position of the contact line perimeter of length m (blue line), and projected area occupied by the vapour phase, S. W' indicates the pinned width of the contact line (in general different to W).

By far the most commonly used approximation for liquid-vapour interfaces in 3D systems is the circular arc model, illustrated in fig. S5(a). For visual clarity we show a reentrant geometry, although the following models and derivations are general for both reentrant and doubly reentrant geometries at all contact angles. In this, a circular arc spans the separation between two adjacent pillars. At the critical pressure for cap failure the arc makes a contact angle of $\theta_\circ^{min}$ with each cap underside. To choose which pillars should be spanned by a circular arc, there exist two choices, shown in fig. S5(b), either the arc spans horizontally separated pillars, or diagonally separated pillars. These are labelled as the horizontal and diagonal models respectively. The distance spanned by the circular arc, $s_\circ$, is therefore equal to $B - W'$ in the horizontal model, or $\sqrt{2}(B - W')$ in the diagonal model. Generally expressed, the radius of curvature, R is

$$R = \frac{s_\circ}{2\sin\theta_\circ^{\min}}, \tag{14}$$

and the sagging height H_c is

$$H_c = \frac{s_\circ}{2}\frac{1-\cos\theta_\circ^{\min}}{\sin\theta_\circ^{\min}}. \tag{15}$$

We emphasise that the sagging height at the onset of cap failure is equivalent to the critical height, as a geometry with a height $H = H_c$ would fail simultaneously via cap failure and base failure.

In fig. S5(c), the circular arc model is tested for accuracy at representing the simulated interface shape. In fig. S5(c)(i) ($W_r = 0.25$), the commonly used diagonal circular arc model (dotted red line) grossly overestimates the sagging height at the critical pressure by several times. The horizontal circular arc model also overestimates the sagging height (dashed red line).It is only at the largest cap widths that the circular arc is able to accurately estimate H_c, as shown for the diagonal model in fig. S5(c)(ii) ($W_r = 0.85$). The reason for this 2D-like behaviour at large W_r, is that the principal radius of curvature approximated by the circular arc (R_1) is significantly smaller than the second principal radius of curvature of the interface (R_2). This arises because $s_\circ << W_r$. Thus, the Laplace pressure $\Delta P \propto 1/R_1 + 1/R_2$ and hence the interface shape, becomes well-approximated by the single radius of curvature R_1.

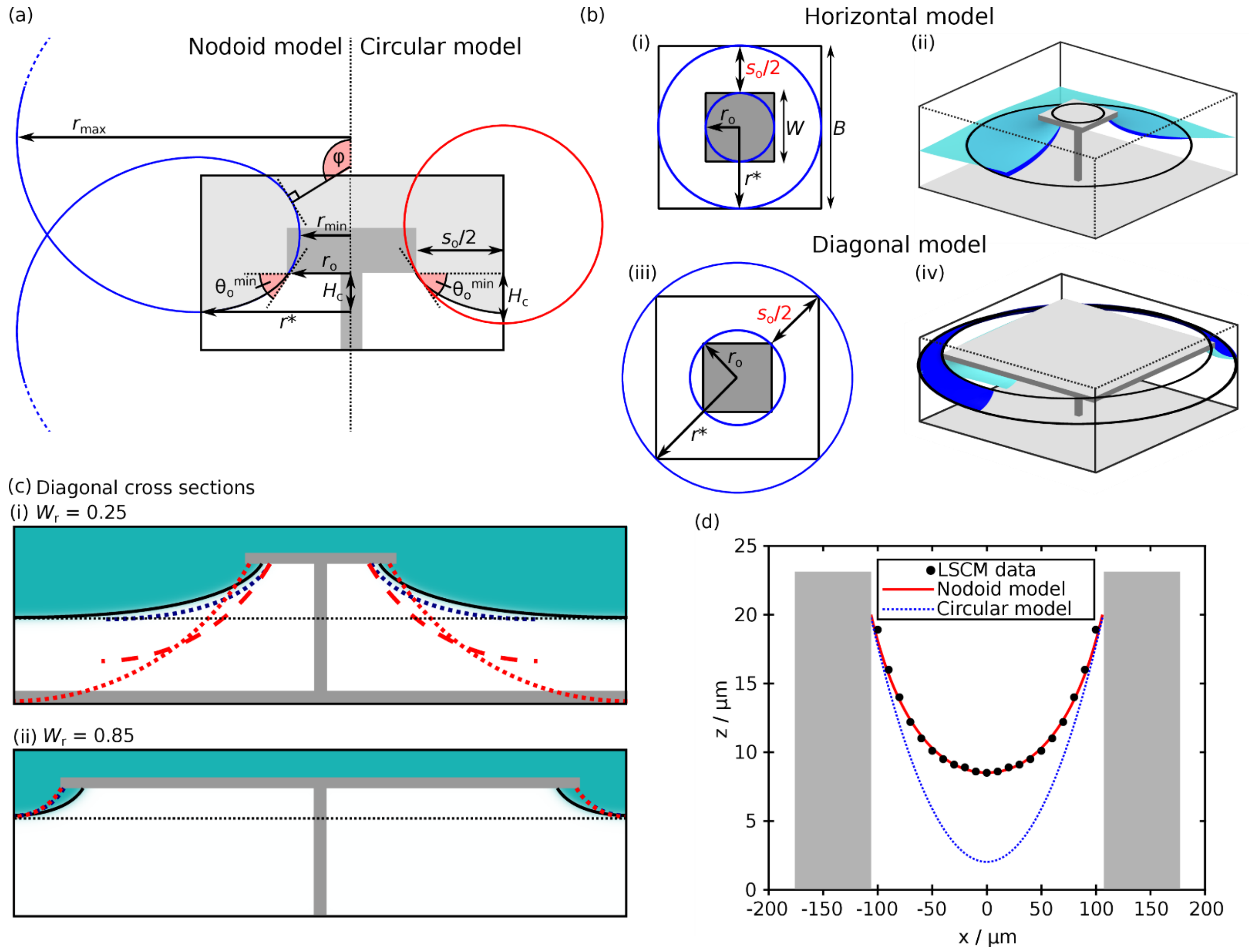

Fig. S5. Analysis and comparison of the circular arc and nodoid models of the sagging depth of the liquid-vapor interface at the critical pressure. (a) Models used to approximate the true liquid-vapour interface morphology (black line). A reentrant geometry is shown for visual clarity, but the models are also valid for doubly reentrant geometries. (Left) The nodoid model for approximating the vapour phase as a capillary bridge (blue line); (right) the circular arc model (red line). All distances and angles used in the model constructions are labelled. (b) In a square system, two choices of distance measurement exist, either horizontal (i,ii) or diagonal (iii,iv). (i,iii) vertical projections of the system, showing inner and outer radii for the nodoid model (blue), and cap separation for the circular model. (ii,iv) Example comparisons with simulations for the horizontal and diagonal models at W_r = 0.25 and W_r = 0.85 respectively. The simulated liquid-vapour interface is shown in light blue, the nodoid model shown in dark blue. Both interfaces are cut horizontally and diagonally to be able to compare the model and simulation results. Black lines indicate the inner and outer perimeters of the nodoid interface. (c) Diagonal cross sections from the 3D horizontal (i) and vertical (ii) models, the sagging height of the liquid-vapour interface obtained from simulation is indicated by a black dotted line. Also shown are the liquid vapour interfaces from: simulation (black, solid), nodoid model (blue, dotted), diagonal circular model (red, dotted), and horizontal circular model (red, dashed, only shown in (i)). In (ii), the diagonal nodoid and circular models overlap, showing how both agree closely with the simulated critical height. (d) Comparison of the diagonal nodoid (red, solid line) and circular arc models (blue, dashed line) with an experimental laser scanning confocal microscopy (LSCM) diagonal profile (black data) of a liquid-vapour interface on a square array of square pillars (grey). Experimental data extracted from (*32*). Note the stretched vertical scale for visual clarity.

However, surfaces with large W_r suffer from a severely limited capacity to produce surfaces of low contact angle hysteresis. For practically useful surfaces where the circular arc model is highly inaccurate, we consider an alternative capillary-bridge model. In this, we recognise that the liquid-vapour interface under the cap forms a capillary-bridge-like structure, in which the vapour has a negative pressure relative to the liquid. We therefore look to model the interface as the simplest 3D surface of constant mean curvature. Out of the family of Delaunay surfaces, the nodoid exhibits a negative mean curvature. This is illustrated in fig. S5(a), in which the blue line shows a portion of interfacial profile of the axisymmetric, periodic surface. In the parametrisation shown, the vertical height z of a point on the surface is defined by the local radius r and angle relative to the surface-normal φ. In the following derivation, we only present the nodoid characteristics pertinent to ascertaining the critical height, for a comprehensive treatment see for example (*52*). The surface is fully characterised by specifying the innermost radius, r_{min}, outermost radius, r_{max}, and a single point on the surface. This point can be determined by realising that, at the critical pressure, the interface makes an angle of π - $\theta_\circ^{min}$ in the vapour phase with the underside of the cap at the pinning location. Thus, we are able to specify the point $(z_\circ, r_\circ, \varphi_\circ) = (H, W'/2, \theta_\circ^{min})$ for the horizontal nodoid model, or $(H, \sqrt{2}W'/2, \theta_\circ^{min})$ for the diagonal nodoid model, shown in fig. S5(b). We are now able to deduce the appropriate r_{min} and r_{max} for each pillar geometry. Firstly, on the portion of the nodoid

representing the liquid-vapour interface, there exists a point at radius $r^* = \sqrt{r_{\min} r_{\max}}$ where $dr/dz = 0$. To respect the periodic boundary conditions of the liquid-vapour interface, $r^* = B/2$ or $r^* = \sqrt{2}B/2$ in the horizontal and diagonal models respectively. Secondly, by rearranging the relationship

$$\begin{aligned} \sin\varphi &= \frac{r^2 - r_{\min} r_{\max}}{r(r_{\max} - r_{\min})}, \\ &= \frac{r^2 - r^{*2}}{r(r^{*2}/r_{\min} - r_{\min})}, \end{aligned} \tag{16}$$

we are able to find $r_{\min}$ as

$$r_{\min} = \frac{1}{2r\sin\varphi}\left[r^{*2} - r \pm \sqrt{(r - r^{*2})^2 + (rr^*\sin\varphi)^2}\right], \tag{17}$$

by taking the negative result and substituting for one of the defined points $(r,\varphi) = (r_\circ,\varphi_\circ)$.

Finally, we are able to obtain the nodoid approximation to the interfacial profile through the relationship

$$z(r) = r_{\min} F(k,\psi) - r_{\max} E(k,\psi), \tag{18}$$

where $$k^2 = 1 - \frac{r_{\min}^2}{r_{\max}^2}, \tag{19}$$

and $$\sin^2\psi = \frac{r_{\max}^2 - r^2}{r_{\max}^2 - r_{\min}^2}, \tag{20}$$

which is plotted in fig. S5(c) (blue dashed line). $F(k,\psi)$ and $E(k,\psi)$ are the elliptic integrals of the first and second kinds respectively. Overall, H_c is obtained through recognising that

$$H_c = z(r_\circ) - z(r^*). \tag{21}$$

Experimental validation of the nodoid model is shown in fig. S5(d). Here, experimental data (black points) are extracted from laser scanning confocal microscopy (LSCM) experiments performed in (*32*), which was able to resolve a diagonal cross-section of the liquid-vapour interfacial profile of a sessile water droplet on a square array of square pillars. For this system, the pillar width $W = 50\ \mu$m, height $H = 23\ \mu$m, system size $B = 200\ \mu$m, and the contact angle is determined to be 109°. As the nodoid and circular arc models only require a knowledge of the contact angle, pillar separation and contact line diameter, both models are equally applicable for simple pillars, reentrant, and doubly reentrant geometries. We compare diagonal variants of both models with the diagonal experimental profile in fig. S5(d). The nodoid model (red, solid line) is observed to closely follow the experimental data, whereas the circular arc model (blue, dashed line) overestimates the sagging height of the interface by 56%.

2.4 Transition mechanisms

2.4.1 Individual mechanisms

To conclude the extended discussion for the reentrant and doubly reentrant geometries at $\theta_\circ = 60°$, we present the energetic barriers for each separate wetting mechanism in fig. S6. For the reentrant geometry, the two possible mechanisms are the Base Contact and Pillar Contact modes, shown in fig. S6(a). For Base Contact, at large pillar heights H_r, the three-phase contact line is no longer able to remain pinned to the base of the cap, and is unstable with respect to depinning and sliding inwards. This places an upper height limit on the existence range of Base Contact, shown in fig. S6(a)(i). This upper height limit decreases with decreasing cap width W_r in a manner similar to the suppressed critical height at low W_r in the critical pressure discussion. In this, the small, negative radius of curvature of the liquid-vapour interface around the cap enforces an even smaller, positive radius of curvature outwards from the cap. The effect of this is to increase the contact angle the liquid-vapour interface makes with the cap underside, meaning that sliding occurs at smaller pillar heights H_r than for structures with larger cap widths W_r.

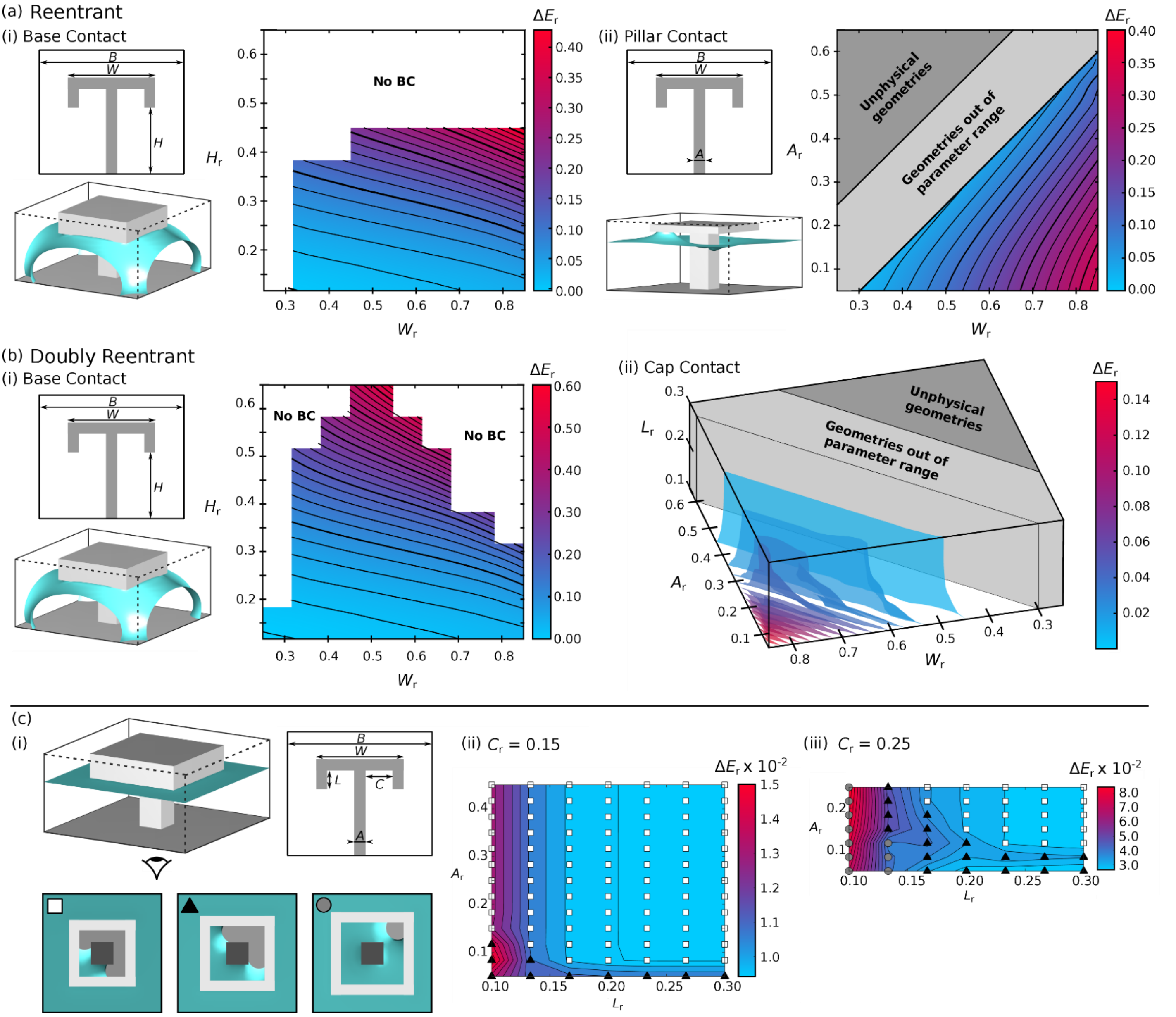

Fig. S6. Effects of the reentrant and doubly reentrant structural parameters on each transition type. (a) 3D illustration and contour plot of the two reentrant transition mechanisms: (i) Base Contact, (ii) Pillar Contact. Impossible geometries with pillar widths A_r wider than the cap width W_r are shaded in dark grey. Geometries approaching this limit and requiring infeasibly large computational domains are shaded in light grey. (b) 3D illustration and contour plot of the two doubly reentrant transition mechanisms: (i) Base Contact, (ii) Cap Contact. (c) Extended description of the cap contact transition mechanism. (i) 3D illustration and under-cap views of the three transition variants (each labelled with a square, triangle, or circle). (ii, iii) Two slices through the 3D contour plot at constant cap width values C_r = 0.15 and C_r = 0.25 respectively. Each datum is labelled with a symbol corresponding to the transition variant shown in (i).

For Pillar Contact on the reentrant geometry, the energetic barrier does not depend on the pillar height H_r, and is therefore the only possible transition mechanism at large pillar heights where Base Contact is inoperative. However, two parameters which do influence the Pillar Contact barrier are the cap width W_r and pillar width A_r. The energetic barrier is increased when the area of the liquid-vapour interface at the transition state is increased. This is observed to occur by maximising W_r and minimising A_r, as shown in fig. S6(a)(ii).

For the doubly reentrant geometry, the two transition mechanisms, Base Contact and Cap Contact, are shown in fig. S6(b). For cap widths W_r < 0.7, the Base Contact mechanism, shown in fig. S6(b)(i), is operative over a greater range of pillar heights H_r than for the reentrant geometry. This is due to the inner cap lip being an effective pinning site, so preventing the liquid-vapour interface from sliding inwards. However, at cap widths W_r > 0.7, it becomes energetically favourable for the liquid-vapour interface to depin from the cap corners, leading to liquid filling the underside of the cap. In these scenarios, Base Contact becomes unstable with respect to the cap-filling, Cap Contact mechanisms.

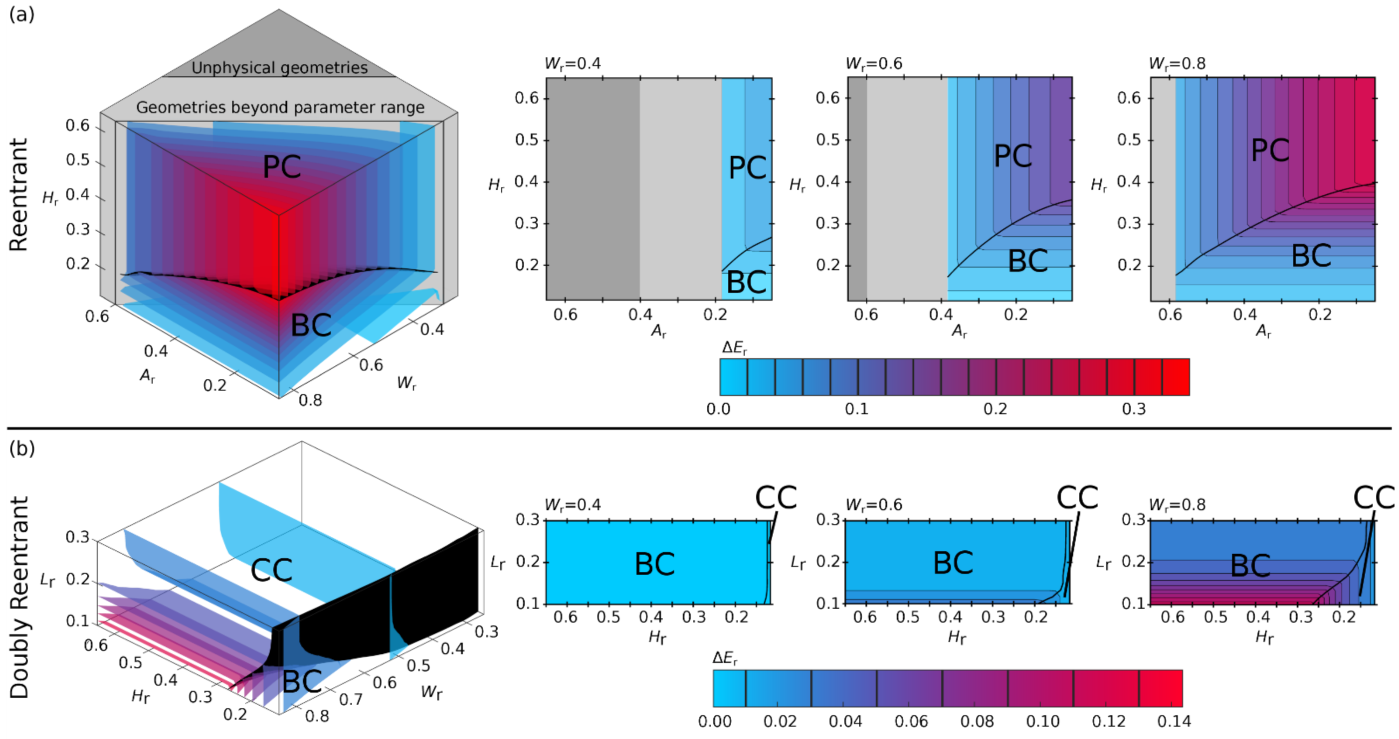

Fig. S7. Extended analysis of the lowest energy reentrant and doubly reentrant transition mechanisms. (a) 3D contour plot of the lowest energy reentrant geometry transition mechanisms: Base Contact, BC; and Pillar Contact, PC. Three slices through the 3D contour plot are also shown for W_r = 0.4, 0.6, and 0.8. Impossible geometries with pillar widths A_r wider than the cap width W_r are shaded in dark grey. Geometries approaching this limit and requiring infeasibly large computational domains are shaded in light grey. (b) 3D contour plot of the lowest energy doubly reentrant geometry transition mechanisms: Base Contact, BC; and Cap Contact, CC. Three slices through the 3D contour plot are also shown for W_r = 0.4, 0.6, and 0.8.

For the doubly reentrant geometry, the Cap Contact mechanism exists over the entire parameter range tested, shown in fig. S6(b)(ii). Because Cap Contact is a heterogeneous condensation mechanism, in which nucleation proceeds from one cap corner, we expect Cap Contact to be operative for all physical doubly reentrant geometries. As with pillar contact on the reentrant geometry, the energetic barrier is increased by increasing the area of the liquid-vapour interface at the transition state. This is shown in fig. S6(b)(ii) to be achieved by maximising the cap width W_r and minimising the pillar width A_r.

However, three mechanistic variants of Cap Contact are observed, shown in fig. S6(c)(i), and their existence ranges are shown in figs. S6(c)(ii,iii). For ease of comparing the transition mechanisms, throughout we have expressed the Cap Contact energy barrier as a function of the cap width W_r. However, the more pertinent parameter for the internal condensing mode is the inner cap width C_r. We show contour plots for the Cap Contact energy barrier at constant C_r = 0.15 and C_r = 0.25 in figs. S6(c)(i) and (ii) respectively. When the internal cavity width C_r is small, at large lip depths L_r and large pillar widths A_r, liquid is most readily able to condense within the cavity as the energetic penalty for forming a liquid-vapour interface is offset early in the transition by forming a large energetically favourable liquid-solid contact area. Thus, under these conditions, the critical nucleus is relatively small (labelled with the white square). As the cavity size C_r increases, the lip depth L_r decreases, or the pillar widths A_r decreases, the energetic offset for forming the liquid-vapour interface is reduced in magnitude. Thus, the critical nuclei occur later in the transition pathway, with larger energy barriers to overcome. These higher-energy critical nuclei are labelled with black triangles, or grey circles in the most extreme cases.

2.4.2 Combined mechanisms

In fig. S7(a,b), the barriers of the lowest energy transition mechanisms are shown for the reentrant and doubly reentrant geometries respectively. For visual clarity, three additional panels are included for each, which represent cuts through the 3D contour plots at constant values of W_r = 0.4, 0.6, and 0.8. For both geometries these panels highlight the increasing barrier with increasing cap width W_r (all other parameters fixed), as well as the competition between the transition mechanisms.

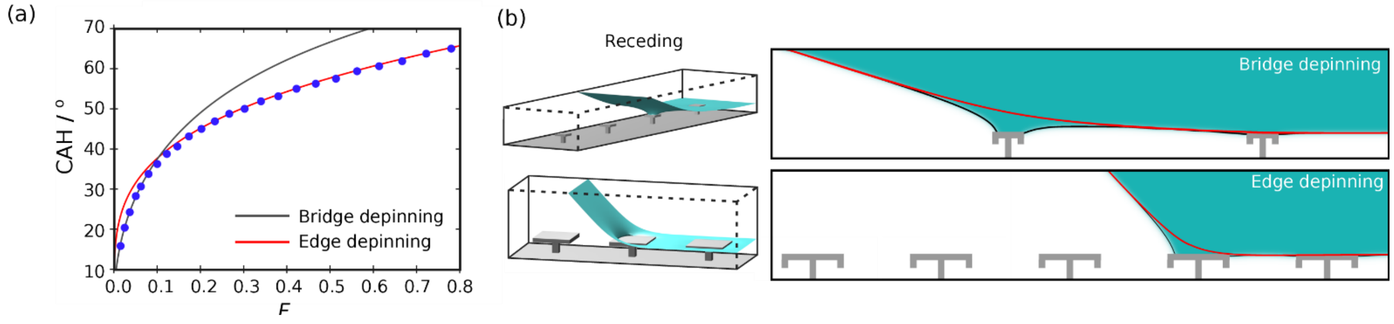

Fig. S8. CAH mechanisms for θo = 110°. (a) Contact angle hysteresis measured via simulation (blue circles), with fitted bridge depinning (grey line) and edge depinning (red line) models. (b) Visualisations of systems at the point of receding via the bridge and edge depinning mechanisms.

2.5 Scoring function sensitivity

Here, we assess the sensitivity of the optimised geometric dimensions and wetting properties to the choice of scoring function parameters for both the membrane distillation and digital microfluidics applications. This is achieved through re-optimising the geometries when each parameter in the scoring functions,

$$
\begin{aligned}
S_{\mathrm{P}} &= \frac{1}{2}\left[1 + \tanh\left(\frac{\Delta P_{\mathrm{c}} - \Delta P_{\mathrm{c}}^{\mathrm{target}}}{\Delta P_{\mathrm{width}}}\right)\right],\\
S_{\mathrm{B}} &= \frac{1}{2}\left[1 + \tanh\left(\frac{\Delta E_{\mathrm{r}} - \Delta E_{\mathrm{r}}^{\mathrm{target}}}{\Delta E_{\mathrm{width}}}\right)\right],\\
S_{\mathrm{C}} &= \max\left(\frac{\mathrm{CAH}^{\mathrm{cutoff}} - \mathrm{CAH}}{\mathrm{CAH}^{\mathrm{cutoff}}}, 0\right),
\end{aligned} \tag{22}
$$

are varied individually by ±5%. For the membrane distillation application, the scoring parameters varied are ΔP_c^{target}, ΔP_{width}, ΔE_r^{target}, ΔE_{width}, and CAH^{cutoff}. On variation of each individual parameter by ±5%, we observe no change in the optimal properties within the resolution to which optimal parameters are determined. The optimised geometry is therefore insensitive to our choice of scoring parameters.

For the digital microfluidics application, the scoring parameters varied are ΔP_c^{target}, ΔP_{width}, and CAH^{cutoff}. Upon scoring parameter variation, the largest change in the optimal geometry is observed in the cap width W_r, originallyoptimised at $W_r = 0.16$. However, then change in cap width is less than ≈5%. The exception to this is when reducing CAH^{cutoff} by 5%, the optimal cap width decreases by 10.5%. The effect of this is to decrease CAH by 13.1% and decrease ΔP_c by 16.7%. The original optimal CAH (25°) is obtained because the linear form of the scoring function S_C favourably weights structures with low CAH. The 5% variation in CAH^{cutoff} (2.5°) represents a large fractional change in CAH, explaining the large change in optimal properties.

Section S3. Results and discussions for θ∘ = 110°

3.1 Contact angle hysteresis

We now consider the wetting properties of non-wetting surfaces. Throughout, the microscopic contact angle is fixed at $\theta_\circ = 110°$.

We begin by surveying the CAH, shown in fig. S8. Because the liquid-vapour interface is pinned to the top of the cap, the CAH is independent of the underlying cap structure. Thus, the reentrant and doubly reentrant geometries show identical behaviour, as do simple posts with no cap structure at all.

The pinning at the top of the cap leads to a uniform advancing contact angle of 180° for all cap widths W_r, matching experimental observations (*53*). Two receding mechanisms are observed, bridge and edge depinning, shown in fig. S8(b). The receding contact angle for the bridge depinning mechanism is described by Eq. (4), where the corrected contact angle $\theta_\circ^{max} = \theta_\circ$ for $\theta_\circ > \pi/2$. Meanwhile, the receding contact angle for the edge-depinning mechanism is described by Eq. (11), in which the term $2D_r$ is neglected as the liquid does not wet the cap sides. In this limit, Eq. (11) reduces to the conventional receding model (*25*). The bridge and edge depinning mechanisms smoothly interpolate on the non-wetting geometries. We find that the error associated with both models compared to the simulation data is minimised if points with $F < 0.1$ are associated with the bridge depinning model, and points with $F > 0.1$ are associated with the edge depinning model. $F = 0.1$ therefore corresponds to the crossover between

analytic models. Overall, for the bridge model, we find α = 0.65 yields a maximum difference in θ_r of 1°. It is interesting to note that here, α < π/4, meaning that the contact line is shorter than a circle of width *W*. Such small values of α have been previously reported (*20*), and suggests that not all portions of the contact line contribute equally to the pinning force, as was our assumption in Eq. (2). However the excellent agreement between theory and simulation allow us to conclude that the proposed capillary bridge model provides a suitable approximation for the actual receding contact angle. The edge-depinning model also shows excellent agreement with the simulation data, in which the maximum difference in θ_r between theory and simulation is also 1°.

3.2 Critical pressures

The critical pressure behaviour observed in fig. S9 is identical for both reentrant and doubly reentrant geometries, as the contact line is always pinned to the outer cap edge. The behaviour is similar to that observed for the doubly reentrant geometry at $\theta_\circ$ = 60° (pinned to the inner cap edge), and would be identical in the limit that the lip width $l \rightarrow 0$. This is because in all three cases, the corrected contact angle in Eq. (13) is $\theta_\circ^{min}$ = 90°. As found previously, the 2D circular arc approximation in Eq. (15) grossly overestimates the critical height for all but the largest cap widths. The 3D capillary bridge models are again shown to be accurate at low area fractions F_r (horizontal model), and high F_r (diagonal model), and correctly capture the qualitative behaviour at intermediate values.

In fig. S9(b), we show the comparison between the critical pressure model for cap failure, expressed in Eq. (13), with the simulation data. As the contact line closely follows the cap edge, excellent agreement between the model and simulation is achieved when α = 1 (square contact line), yielding a fitted a = 0.02. As anticipated, because the contact line is pinned to the outer cap edge, a is insignificantly different from zero, relative to the interface width = 0.01.

3.3 Minimum energy barriers

For $\theta_\circ$ = 110°, Base Contact and Pillar Contact mechanisms are observed, illustrated in fig. S10(a,b). A video of the Pillar Contact mechanism is shown in Movie S4, while the Base Contact mechanism exhibits a similar pathway to that shown in Movie S1. Throughout, there is negligible difference between the wetting mechanisms for reentrant and doubly reentrant geometries. Both the Base Contact and Pillar contact mechanisms are similar to the $\theta_\circ$ = 60° cases. However for Pillar Contact, as illustrated in fig. S10(c), the energy barrier (*) occurs much later in the reaction pathway for $\theta_\circ$ = 110° (black line) than for $\theta_\circ$ = 60° (red dashed line). Despite the complex interface morphologies, a simple relationship is derived to predict which Pillar Contact variant is operative for a given set of structural parameters. If the total energy of the system is lowered by the liquid-vapour interface sliding down the pillar, the minimum energy barrier occurs early in the transition pathway. This occurs prior to sliding when the liquid contacts the cap, as observed at $\theta_\circ$ = 60°. Otherwise, the minimum energy barrier will occur late in the transition, after the sliding process, and immediately before the interface contacts the base of the system, as observed for $\theta_\circ$ = 110°. Such a mechanism was originally proposed to give rise to the barrier to the Cassie-Baxter state on superhydrophobic surfaces *(54).* More generally, we may consider the energy change of an interface sliding down the pillar at constant interface shape. If

$$\Delta P_{\mathrm{r}} > \frac{4\cos\theta_\circ}{A_{\mathrm{r}} - \frac{1}{A_{\mathrm{r}}}}, \tag{23}$$

sliding is energetically favourable, and the energy barrier will occur early in the transition mechanism. As we restrict ourselves here to the study of transitions at zero pressure, this explains why the early barrier is observed for $\theta_\circ$ < 90°, whilst the late transition is observed for $\theta_\circ$ > 90°.

The Pillar Contact mechanism is further observed to have three variants, illustrated in fig. S10(b). The variant is selected based on the pillar width A_r, such that for type (i), $A_r \leq 0.12$, for type (iii), $A_r \geq 0.22$, and type (ii) occurs at intermediate values.

We now consider the minimum energy barrier as a function of the structural parameters. For Base Contact, shown in fig. S10(d), the liquid-vapour interface morphology depends on the pillar height H_r and cap width W_r. As the interfacial area is maximised at large H_r and W_r, the largest minimum energy barriers occur under these conditions. However, upon increasing the height, there exist heights above which the contact line can no longer remain pinned to the outer cap edge, meaning that Base Contact is prevented. For non-wetting liquids, the suspended free energy minimum sees the interface pinned to the top of the cap (at zero applied pressure), whereas the liquid must wet the entire sides of the cap to overcome the minimum energy barrier. Thus, in contrast to $\theta_\circ$ = 60°, at $\theta_\circ$ = 110°, the minimum energy barrier is increased by increasing the total lip depth D_r.

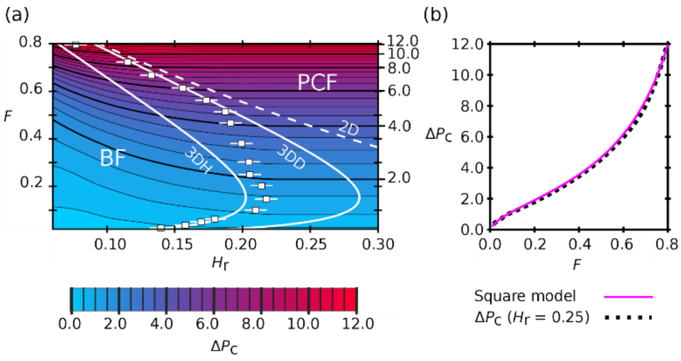

Fig. S9. Critical pressure analysis for θo = 110°. (a) Critical pressure variation with area fraction F_r and pillar height H_r. Data points indicate the critical height, and therefore the boundary between Base Failure BF, and pinned Cap Failure PCF. Error bars represent the interface width, and hence the uncertainty in the critical height. Approximations to the critical heights are indicated for circular 2D model (white, dashed line), and both horizontal and diagonal nodoid variants (white, solid line). (b) Comparison of the cap failure model against the simulation data for $H_r = 0.25$.

In the Pillar Contact mechanism, shown in fig. S10(e), the liquid-vapour interface morphology at the minimum energy barrier is affected only by the pillar width A_r. However, during the transition, almost the entire solid area of the reentrant or doubly reentrant pillar becomes wetted, yielding a concomitant energetic penalty. Therefore, the minimum energy barrier depends on all structural parameters, but in fig. S10(e) we choose to fix $D_r = 0.15$. Although the doubly reentrant cap structure has a larger surface area than the reentrant cap, for the parameter ranges considered here this only marginally impacts the minimum energy barrier.

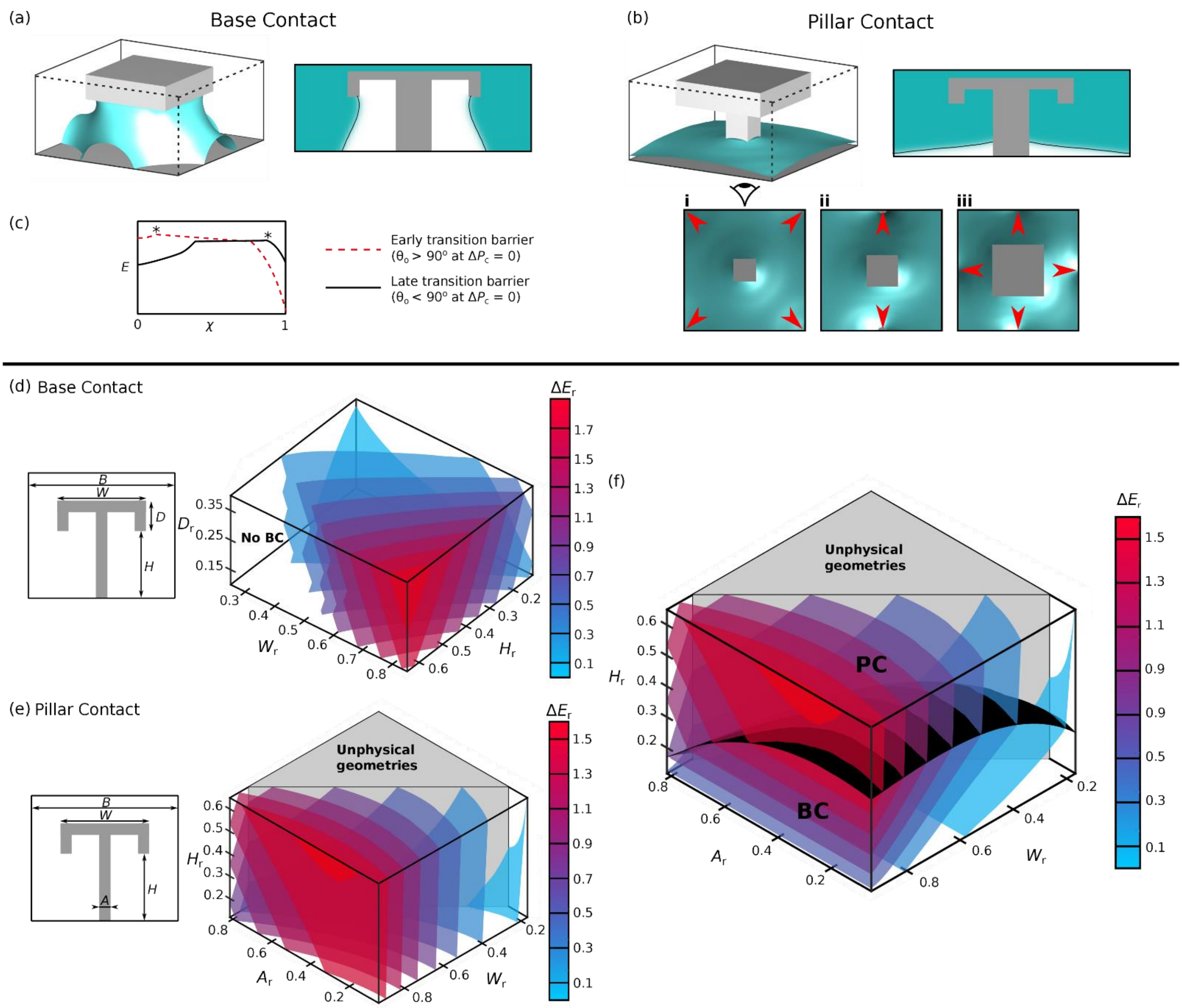

Fig. S10. Individual and lowest energy transition mechanisms for θo = 110°. (a) 3D visualisation and 2D diagonal cross sections of a representative Base Contact example. (b) 3D visualisation and 2D diagonal cross section a representative Pillar Contact example, in which the under-cap views shown the three symmetric variants. Red arrows indicate the positions where the liquid-vapour interface first contacts the base of the system. (c) Illustration of the two forms of Pillar Contact energetic profiles *E* as a function of the reaction coordinate *s*, with barriers labelled with a *. *s* = 0 in the suspended state and 1 in the collapsed state. (d-f) 3D contour plots showing the minimum energy barrier for Base Contact (d) and Pillar Contact (e), and the minimum energy transition path (f) as a function of the height H_r, cap width W_r, pillar width A_r, and total lip depth D_r. Markings on the colour bars indicate the energies of each constant-barrier surface.

Finally, over the range of structural parameter values, we evaluate the minimum energy transition mechanism, shown in fig. S10(f). In this, we fix D_r = 0.15. At the lowest pillar heights, the Base Contact mechanism is operative, but changes to Pillar Contact once the energetic penalty for forming the large liquid-vapour interface in Base Contact becomes too large. Unlike in the $\theta_\circ$ = 60° case, here the barrier can be increased indefinitely by increasing the total solid area of the structure which is wetted during the transition. This can be achieved effectively through extending H_r or D_r.

Movie S1. BC mechanism for a doubly reentrant geometry at θ_o = 60°. (N_x, N_y, N_z) = (60, 60, 50), (A_r, H_r, W_r, t_r, L_r, l_r) = (0.05, 0.23, 0.45, 0.05, 0.10, 0.05). Shown is a 3D visualisation of the liquid-vapour interface, 2D diagonal cut through the system showing the local order parameter ϕ, and the energy profile along the transition pathway as a function of the normalised path length *s*.

Movie S2. PC mechanism for a reentrant geometry at θ_o = 60°. (N_x, N_y, N_z) = (60, 60, 50), (A_r, H_r, W_r, t_r) = (0.05, 0.33, 0.68, 0.05). Shown is a 3D visualisation of the liquid-vapour interface, 2D diagonal cut through the system showing the local order parameter ϕ, and the energy profile along the transition pathway as a function of the normalised path length *s*.

Movie S3. CC mechanism for a doubly reentrant geometry at θ_o = 60°. (N_x, N_y, N_z) = (60, 60, 50), (A_r, H_r, W_r, t_r, L_r, l_r) = (0.05, 0.23, 0.45, 0.05, 0.10, 0.05). Shown is a 3D visualisation of the liquid-vapour interface, 2D diagonal cut through the system showing the local order parameter ϕ, and the energy profile along the transition pathway as a function of the normalised path length *s*.

Movie S4. PC mechanism for a reentrant geometry at θ_o = 110°. (N_x, N_y, N_z) = (60, 60, 50), (A_r, H_r, W_r, t_r) = (0.05, 0.33, 0.68, 0.05). Shown is a 3D visualisation of the liquid-vapour interface, 2D diagonal cut through the system showing the local order parameter ϕ, and the energy profile along the transition pathway as a function of the normalised path length *s*.
